# Invasive alien mammals pose zoonotic risks to human health in Europe

**DOI:** 10.1016/j.onehlt.2025.101307

**Published:** 2025-12-19

**Authors:** Paola Monguilod, Belinda Gallardo

**Affiliations:** a University of Zaragoza, C. de Pedro Cerbuna, 12, 50009 Zaragoza, Spain; b Pyrenean Institute of Ecology, Av. de Montañana, 1005, 50059 Zaragoza, Spain

**Keywords:** Invasive alien species, Invasive mammals, Zoonoses, Climate change, Species distribution models, Disease hotspots, Emerging infectious diseases

## Abstract

The rise in zoonotic diseases is accelerating, with climate change expected to further intensify this trend. Invasive Alien Species (IAS) play an important role in the emergence and spread of zoonotic diseases by introducing both existing and novel pathogens to the regions they invade. Despite this, research on the role of IAS in spreading zoonotic diseases remains limited. Our study investigated the zoonotic risks posed by eight invasive mammal species prioritized for management in Europe. On average, each species was found to transmit 16 pathogens capable of causing severe diseases in humans, including Echinococcosis, Leptospirosis, Lyme neuroborreliosis, and Encephalitis.

We identified central and western Europe as significant disease hotspots. Climate change is facilitating the expansion of IAS into new areas, as warmer temperatures make previously inhospitable regions suitable. Future projections indicate a northeastward shift in their suitability by 2050. These changes vary by species, with the Siberian chipmunk losing up to 45 % of its suitability, while the gray squirrel could see a 26 % increase under a high-emissions scenario.

Finally, we found that 71 % of the human population lives in areas highly suitable for IAS establishment. Our findings underscore the health risks associated with IAS and highlight the need for further research into their role in disease dynamics. Addressing this issue is essential for developing effective public health strategies and mitigating future zoonotic disease outbreaks.

## 1 Introduction

Zoonotic diseases from wildlife represent over 40 % of emerging infectious disease (EID) events globally since 1940, posing significant threats to human health [25]. While current responses focus on outbreak containment, early detection of new pathogens remains challenging [1]. Invasive Alien Species (IAS) are increasingly recognized as contributors to zoonotic disease emergence due to their role as pathogen reservoirs, rapid expansion in human-modified environments, and disruption of native ecosystems [35,44].

IAS often carry pathogens novel to the invaded ecosystem and proliferate due to the absence of natural predators [14]. IAS are introduced and proliferate in human-modified environments where animal-human contact is frequent [28]. This increases the risk of zoonotic transmission, as demonstrated by the Nipah virus outbreak in Malaysia, where deforestation followed by intensive pig farming created ecological conditions that enabled the virus to spill over from fruit bats to pigs and subsequently to humans [30].

IAS contribute to zoonotic disease emergence globally. For example, in Cuba, the giant African snail (*Achatina fulica*) has raised the risk of eosinophilic meningitis in humans by hosting *Angiostrongylus cantonensis* [29].

Mammals, due to their phylogenetic proximity to humans, are especially likely to transmit zoonoses [44].

Rising temperatures associated with climate change have been shown to facilitate both the spread of IAS and infectious diseases, especially those transmitted by vectors such as mosquitoes [10,15,31,34]. However, climate change involves not only higher temperatures but also prolonged warm periods, altered precipitation patterns, and increased climatic variability, all of which can further influence the distribution of IAS and the emergence of zoonotic diseases. Despite this, the combined impacts of climate change on both IAS and zoonotic disease emergence remain underexplored.

This study investigates how invasive mammals in Europe contribute to zoonotic disease emergence. Europe provides a well-documented context, given its long history of mammal introductions, established regulatory frameworks (e.g., EU Regulation No. 1143/2014), and high human population density [9,40].

To achieve this, the following specific objectives are proposed: (i) identify invasive mammals capable of transmitting zoonotic diseases in Europe; (ii) determine the pathogens they carry that are significant for human health; (iii) spatially identify the areas at greatest risk of invasion and thus of EIDs transmission, called “disease hotspots”, and calculate the exposure of the human population; and (iv) project changes in invasive mammal distribution and human population exposure under 2050 climate change scenarios.

Though focused on Europe, the approach provides a framework that can be applied to other regions to assess zoonotic risks associated with IAS.

## 2 Methods

This study followed a structured approach to assess the zoonotic risk associated with invasive mammals in Europe (see Suppl. Fig. 1): (1) identifying invasive mammals of concern; (2) characterizing the zoonotic pathogens they carry; (3) modeling their current and future distribution, mapping potential disease hotspots and estimating human exposure.

### 2.1 Step 1: Identify invasive mammals of concern in Europe

From the 11 invasive mammals listed in the EU Union List, eight were selected based on evidence of their potential as zoonotic pathogen reservoirs, as reviewed in Roy et al. [35]. For each mammal, we collected information about habitat, origin, introduction pathway, and first records in Europe.

### 2.2 Step 2. Characterize the main pathogens carried by invasive mammals

Zoonotic pathogens associated with the eight selected invasive mammals were identified using literature from Roy et al. [35], EU risk assessments, and a targeted search of primary studies on Google Scholar (March 2023) using each species' scientific name with the term “zoonotic”, screening 10 articles per species.

During this literature review, we established specific criteria for inclusion and exclusion of the articles initially retrieved, ensuring that they were in line with the scope of our study (modified from [35]; Table 1).

**Table 1:** Inclusion and exclusion criteria used to select studies for the review.

Inclusion Criteria	• Contains primary data on populations of IAS causing (or having potential to cause) zoonotic disease. • Contains primary data on the role of IAS (or potential role) as a vector or reservoir species for a zoonotic disease. • Reviews the role of IAS in zoonotic disease transmission and spread in the wild. • Contains primary laboratory data of IAS which are vectors or reservoirs for a zoonotic pathogen.

Exclusion criteria	• Contains only ecological, taxonomic, genetic or physiological data on the IAS with no data on a zoonotic disease. • Contains data on bites by an IAS as a health problem rather than infectious zoonotic disease. • Reviews that do not explicitly link IAS and zoonotic diseases (e.g., of invasion and biosecurity policy, zoonotic diseases and ecosystems, zoonotic diseases and biogeography, wildlife trade) • Paper not in the English language or lacking an abstract.

These criteria were applied to both our own search results and the articles included in Roy et al. [35]. A total of 84 articles met the inclusion criteria. The selection process and the number of articles retained per species are illustrated in Supplementary Fig. 2, and the full list of included articles is provided in Supplementary Table 1.

Priority was given to pathogens shared among multiple mammals, while broadly distributed ones like rabies were excluded. Data were collected on pathogen prevalence, severity, and incidence.

#### 2.2.1 Prevalence data

Pathogen prevalence was gathered from the selected studies (Table S1). Due to limited European data, we also included prevalence estimates from other regions, following the precautionary principle.

When multiple prevalence values for a specific pathogen were available in a single article, we prioritized the most recent and those with a sample size greater than one. If diverse prevalence values were still present, an average prevalence was calculated.

Prevalence values were recorded as percentages (cases per 100 individuals). Reliability of prevalence sources was classified as:

• **High**: sample size >20 individuals,
• **Medium**: 10–19 individuals,
• **Low**: < 10 individuals.

Pathogens with only low-reliability prevalence data were excluded from further analysis.

#### 2.2.2 Severity data

Pathogen severity was classified using Biosafety Level (BSL) standards [12,47], that designates the level of containment required for handling microorganisms and biological materials in a laboratory.

• **BSL-1**: non-pathogenic or minimal risk.
• **BSL-2**: treatable human pathogens, low transmission risk.
• **BSL-3**: serious diseases, treatable, high spread risk.
• **BSL-4**: serious, untreatable, and highly transmissible diseases.

When pathogens from the same genus or family had varying BSLs, the highest BSL was used. We focused only on pathogens rated BSL-3 or BSL-4.

#### 2.2.3 Incidence in Europe

We obtained incidence data (i.e., the number of reported human cases) for each pathogen in Europe from 1950 to 2023, using the TESSy database managed by the European Centre for Disease Prevention and Control (ECDC) (https://www.ecdc.europa.eu/en).

### 2.3 Step 3: Spatial analysis of the zoonotic risk

Species Distribution Models (SDMs) were used to predict the potential current and future geographic distribution of the selected invasive mammals across Europe (Fig. S1). SDMs relate species occurrence with environmental conditions, estimating the probability of establishment [20,21].

#### 2.3.1 Species occurrence data

We imported global occurrence records from the Global Biodiversity Information Facility database (GBIF, https://www.gbif.org) into R and cleaned them using the ‘scrubr' R package v 0.3.2. [13]. We included additional occurrences from the European Alien Species Information Network (EASIN; https://easin.jrc.ec.europa.eu/easin), and from literature sources [5,17]. To reduce spatial sampling bias, occurrences were spatially thinned to one point per 10 × 10 km grid cell [37].

#### 2.3.2 Predictors used

We incorporated ten environmental predictors previously identified as effective for modeling the distribution of the target invasive mammals [18,32], while ensuring low multicollinearity by excluding variables with high correlation (Pearson's *r* > 0.7) or high Variance Inflation Factor (VIF > 5). The final set of predictors (Table S2) included:

• **Seven bioclimatic variables** (from CHELSA, Climatologies at High resolution for the Earth's Land Surface Areas, version 2.1; [27])
• **Accessibility**, as a proxy for propagule pressure to reflect the strong human influence in the transportation and establishment of IAS [17,19,45].
• **Elevation**, reflecting topographic and climate gradients [2].

All variables were aggregated to a 5 arc-minute resolution (∼10 × 10 km) using the terra R package [22], aligning with the resolution of species occurrence data.

#### 2.3.3 Model calibration

Models were calibrated incorporating data from both native and invasive ranges to encompass all potential environmental conditions for survival. Calibration was performed in R software version 4.2.2 using the BIOMOD2 v4.2–3 package [41], employing an ensemble modeling approach combining four algorithms: Generalized Linear Models (GLM), Generalized Additive Models (GAM), Random Forest (RF), and Boosted Regression Trees (GBM).

As these algorithms require both presence and absence data [3], we generated 10,000 pseudo-absences per species with a 0.5 prevalence to balance training datasets [7]. Each model was trained on 70 % of the data and tested on the remaining 30 %, repeated three times to address uncertainty in partitioning [41], resulting in 12 model replicas per species (4 algorithms × 3 partitions). This procedure relied on random cross-validation, a commonly used approach in SDMs, in which training and testing subsets are created through random partitioning of the occurrence data.

Model performance was evaluated using the True Skill Statistic (TSS), with higher values indicating higher predictive capability. Only replicas with TSS ≥ 0.7 were retained for ensemble modeling to ensure that only the best-performing models were considered for the final ensemble [41].

Ensemble models were obtained by combining all model replicas retained after quality filtering (TSS ≥ 0.7). The ensemble prediction was generated using BIOMOD2's weighted-mean approach, in which each individual model contributes proportionally to its predictive capacity (TSS). This procedure increases model robustness by integrating the strengths of multiple algorithms while reducing individual model uncertainty.

#### 2.3.4 Projections

Ensemble models were projected onto Europe creating continuous suitability maps (0–1000 scale), which were then binarized using the maxTSS threshold [7].

Future projections for 2050 were generated by applying the calibrated ensemble models to the CHELSA climate layers for 2050 under three scenarios (ssp126, ssp370 and ssp585; [26]; https://chelsa-climate.org/; Table S3). In BIOMOD2, projections are produced by transferring the species–environment relationships established during model calibration onto the future climate predictors, while keeping all model parameters constant.

Elevation and accessibility were assumed constant across future scenarios, given the absence of projected data, based on the assumption that these factors will remain similar to their current state [17].

#### 2.3.5 Species range change (SRC)

We assessed range shifts under climate change by comparing current and future binary maps. Areas were categorized as:

• **Loss**: currently suitable, projected to become unsuitable.
• **Gain**: currently unsuitable, projected to become suitable.

SRC maps were produced using QGIS v3.30.3 (https://www.qgis.org/).

#### 2.3.6 Disease hotspot maps

Disease hotspot maps were generated by aggregating continuous suitability maps of all invasive mammals capable of transmitting a given pathogen. For each pathogen, we summed the suitability values of all relevant host species, weighting each species' contribution by the prevalence of that pathogen (see Step 2.1). This results in a series of continuous maps of relative transmission risk, where higher values represent areas with an elevated potential risk of zoonotic disease transmission to humans. Where possible, georeferenced pathogen data from GBIF were overlaid for reference. However, data limitations restricted direct SDM calibration for pathogens. This limitation reflects a broader issue in zoonotic research: GBIF and similar repositories often contain detailed data on hosts and vectors but rarely on pathogens themselves [6].

#### 2.3.7 Estimating human exposure

Human exposure to zoonotic risk was estimated by overlaying invasive mammal distribution maps with population data. Current population data were derived from the Gridded Population of the World v4 dataset [11], while future population projections were obtained from the 1-km downscaled population grids for the Shared Socioeconomic Pathways (SSPs) [24]. Exposure was calculated as the percentage of people living in areas suitable for each mammal under current and future scenarios.

Our analysis aimed to identify broad areas of elevated risk for human-wildlife interactions and potential disease transmission. It is important to note that living in the same 10 × 10 km grid cell as a particular invasive mammal does not necessarily lead to a zoonotic event, which is why we refer to this situation simply as exposure.

## 3 Results

### 3.1 Invasive mammals of concern in Europe

We identified eight invasive alien mammal species with zoonotic potential (Table 2). Most originated from Asia and America and were introduced in the 19th–20th centuries. Introduction pathways included deliberate releases into nature (for biological control or hunting), unaided natural dispersal, and escapes from confinement (from fur farms, botanical gardens, zoos, aquaria, and the pet/aquarium/terrarium trade). Most species subsequently spread widely across Europe. Information on introduction history is taken from the EU risk assessments, and some species had multiple introduction pathways (see Table 2 for details).

**Table 2:** Invasive mammals regulated in Europe and selected for this study, including information on their origin, pathway of introduction and first report in Europe. Also included, the number of published articles about zoonotic risks associated with each species that were found and the total number of pathogens they host. More details in Table S1.

Scientific name (common name)	Order	Origin	Pathway	First introduction in Europe	Num. articles on disease transmission	Num. of pathogens
*Herpestes javanicus* (The Javan mongoose)	Carnivora	Asia	Release in nature: biological control	1910 (Croatia)	5	5
*Myocastor coypus* (Coypu)	Rodentia	South America	Escape from confinement: fur farms. Release in nature. Unaided: natural dispersal	1882 (France)	11	15
*Nasua nasua* (Ring-tailed coati)	Carnivora	South America	Escape from confinement: botanical garden/zoo/aquaria	2003 (United Kingdom)	10	10
*Nyctereutes procyonoides* (Raccoon dog)	Carnivora	Asia	Release in nature: hunting Unaided	1926 (Russian Federation)	18	32
*Ondatra zibethicus* (Muskrat)	Rodentia	North America	Escape from confinement: fur farms Unaided	1905 (Czechia)	12	23
*Procyon lotor* (Raccoon)	Carnivora	North and Central America	Escape from confinement: botanical garden/zoo/aquaria. Release in nature: hunting Unaided	1927 (Germany)	20	21
*Sciurus carolinensis* (Gray squirrel)	Rodentia	North America	Escape from confinement: botanical garden/zoo/aquaria Release in nature.	1876 (United Kingdom)	7	16
*Tamias sibiricus* (Siberian chipmunk)	Rodentia	Asia	Escape from confinement: pet/aquarium/terrarium Release in nature.	1957 (Austria)	3	1

### 3.2 Main pathogens carried by invasive mammals

A total of 97 pathogens were identified across the chosen mammals (average: 16 per species). *N. procyonoides* hosted the most (32), and *T. sibiricus* the fewest (3) (Table 2; Table S1). Six pathogens were selected for further analysis due to their prevalence and severity: *Borrelia burgdorferi s.l.*, *Echinococcus multilocularis*, *Leptospira* spp., *Francisella tularensis*, Tick-borne encephalitis virus, and *Hantavirus* (Table 3). These range in impact from mild symptoms to severe neurological damage.

**Table 3:** Diseases caused by pathogens carried by the eight focus invasive mammals. We chose pathogens with Biosecurity level (BSL) of 3 and 4, indicating that they cause severe diseases with a high risk of spread, with or without available treatment, respectively. Incidence in Europe reflects the total number of disease cases in humans recorded between 1950 and 2023. Prevalence, expressed as percentage of individuals carrying the pathogen, corresponds to the maximum of the range of prevalences reported in literature (Table S1).

Disease	Pathogen causing the disease	Severity (BSL level)	Incidence in Europe (num. cases)	Reservoir Invasive Species	Prevalence
Echinococcosis	*Echinococcus multilocularis*	3	1122	*Myocastor coypus*	0.4 %
				*Nyctereutes procyonoides*	12 %
				*Ondatra zibethicus*	11 %
Leptospirosis	*Leptospira*	3	1544	*Herpestes javanicus*	8 %
				*Myocastor coypus*	38 %
				*Procyon lotor*	30 %
Tularemia	*Francisella tularensis*	4	853	*Nyctereutes procyonoides*	13 %
				*Ondatra zibethicus*	33 %
				*Procyon lotor*	0.5 %
Tick-Borne Encephalitis	*Tick-Borne Encephalitis virus*	3	1098	*Sciurus carolinensis*	3 %
Hantavirus infection	*Hantavirus*	3	719	*Ondatra zibethicus*	8 %
Lyme neuroborreliosis	*Borrelia burgdorferi* Sensu Lato	3	317	*Sciurus carolinensis*	12 %
				*Tamias sibiricus*	35 %

### 3.3 Spatial analysis of zoonotic risk in Europe

#### 3.3.1 Calibration of species distribution models

From over 1 million global occurrence points, 35,531 were retained after the cleaning protocol (Table 4; Table S4).

**Table 4:** Total number of occurrence records used for calibration for each mammal species under study after the cleaning protocol.

Species	Global Occurrence records used for calibration
*Herpestes javanicus*	310
*Myocastor coypus*	5277
*Nasua nasua*	618
*Nyctereutes procyonoides*	2563
*Ondatra zibethicus*	8732
*Procyon lotor*	10,788
*Sciurus carolinensis*	6263
*Tamias sibiricus*	980

Distribution models showed high accuracy (TSS 0.71–0.91; Table S5), with additional evaluation metrics in Table S6.

Key habitat suitability predictors included annual mean temperature (bio1), temperature seasonality (bio4), and human accessibility. Variable importance is detailed in Tables S7–S15 and Figs. S3–S4.

**Fig. 1:**
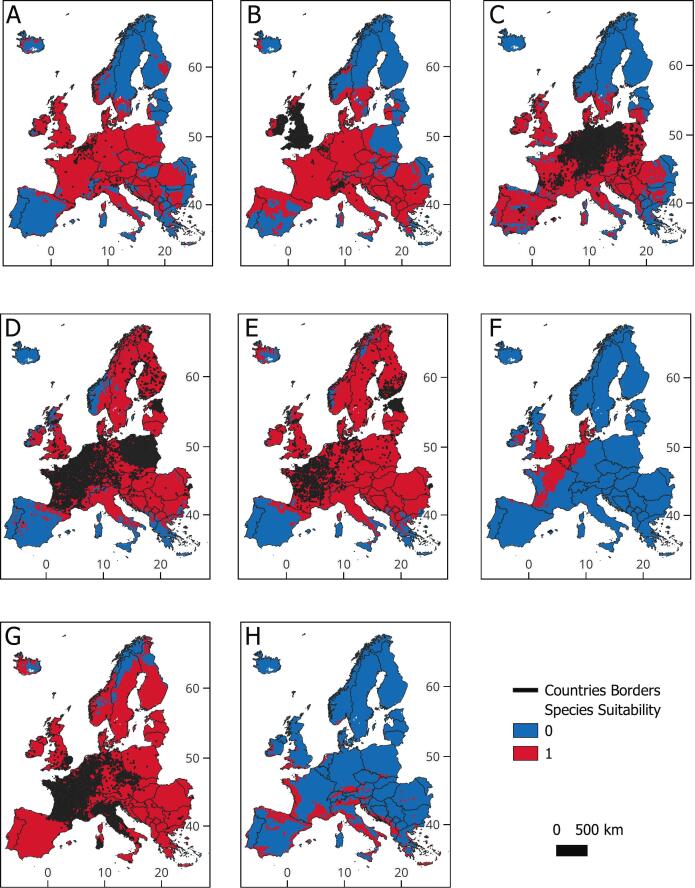
Binary maps displaying the current predicted suitability for eight invasive mammals regulated in Europe. A. *Tamias sibiricus*, B. *Sciurus carolinensis*, C. *Procyon lotor*, D. *Ondatra zibethicus*, E. *Nyctereutes procyonoides*, F. *Nasua nasua*, G. *Myocastor coypus*, H. *Herpestes javanicus*. Blue (0) indicates areas with low suitability for the invasive mammals, where they are unlikely to establish. Red (1) indicates areas very suitable, that is, similar to those inhabited by the invasive mammals, where the species are likely to survive if introduced. Real species occurrences, used to calibrate the models, are represented as black dots. (For interpretation of the references to colour in this figure legend, the reader is referred to the web version of this article.)

Current suitability maps (binary: Fig. 1; continuous: Fig. S5) highlight Western and Central Europe, particularly around the British Channel region (including northern France, southern England, Belgium and nearby coastal regions), as hotspots for the invasive mammals.

#### 3.3.2 Range change under climate change

**Fig. 2:**
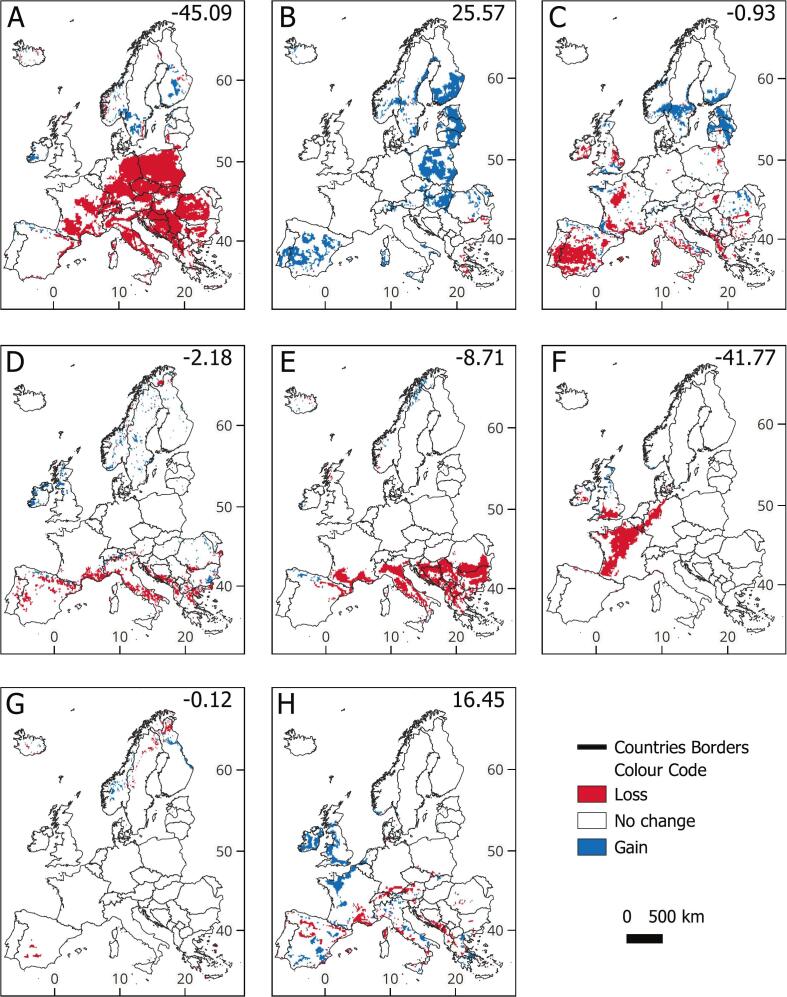
Maps displaying changes in the future suitability for IAS in Europe under the High Emissions scenario compared to the current scenario. Species depicted: A. *Tamias sibiricus*, B. *Sciurus carolinensis*, C. *Procyon lotor*, D. *Ondatra zibethicus*, E. *Nyctereutes procyonoides*, F. *Nasua nasua*, G. *Myocastor coypus*, H. *Herpestes javanicus*. In the top right corner, we display species range change (SRC) as the percentage area gained or lost by 2050 relative to the current scenario.

Species range changes (SRCs) were consistent across the three future scenarios; results for the high emissions scenario are shown (Fig. 2; others in Figs. S6–S7).

Under climate change, habitat suitability shifts northward. Most mammals experience a net range loss (e.g., *T. sibiricus*, −45 %, Fig. 2A), except for *S. carolinensis* (+26 %, Fig. 2B) and *H. javanicus* (+16 %, Fig. 2H), which gain range.

#### 3.3.3 Disease hotspot maps

**Fig. 3:**
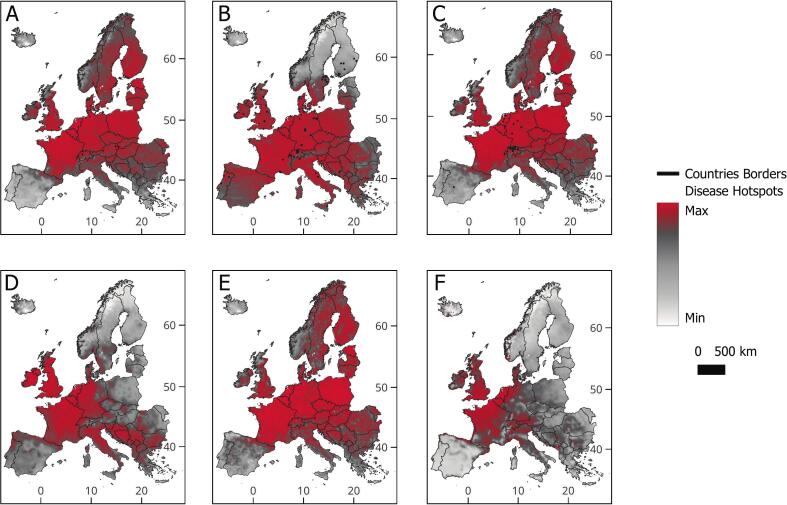
Continuous maps displaying disease hotspots in Europe. Diseases depicted: A. Echinococcosis, B. Leptospirosis, C. Tularemia, D. Tick-Borne Encephalitis, E. Hantavirus infection, F. Lyme neuroborreliosis. Black dots represent georeferenced locations of the pathogens that cause the diseases.

Disease hotspot maps (Fig. 3) reveal regions at elevated zoonotic risk. Central Europe (including Germany, Poland, the Czech Republic, Hungary, Austria, and Slovakia) shows multiple hotspots, especially for Echinococcosis, Leptospirosis, Tularemia, and Hantavirus. Germany presents a heightened risk for the spread of all diseases except Lyme neuroborreliosis. In Southern Europe, Leptospirosis is most prevalent, with Italy particularly affected.

#### 3.3.4 Human exposure to zoonotic risks

Human exposure was estimated as the percentage of the European population residing in areas suitable for each invasive mammal under current and future scenarios (Table 5).

**Table 5:** Exposure of the European human population to invasive mammals. The current scenario represents the % of inhabitants in Europe that inhabit areas highly suitable for the establishment of invasive mammals, under the current climate conditions. Future scenarios represent the increase or decrease (in % relative to the current scenario) in the human population inhabiting areas suitable for the invaders.

	Current scenario	Low emissions scenario	BAU scenario	High emissions scenario
*Herpestes javanicus*	22,50	+38 %	+21 %	+33 %
*Sciurus carolinensis*	82,43	+10 %	+10 %	+12 %
*Myocastor coypus*	99,43	0 %	0 %	0 %
*Procyon lotor*	81,80	+1 %	-9 %	−7 %
*Ondatra zibethicus*	85,20	−5 %	−6 %	−6 %
*Nyctereutes procyonoides*	87,49	−7 %	−12 %	−13 %
*Nasua nasua*	28,80	+5 %	−14 %	−30 %
*Tamias sibiricus*	78,16	−23 %	−63 %	−40 %

The Javan mongoose and gray squirrel showed the largest projected increases, while the coypu already had the highest exposure (99 %) with minimal change expected. Exposure to the other five species is expected to decline by 5–63 %.

## 4 Discussion

Despite growing recognition of the role of Invasive Alien Species (IAS) in disease transmission, their impacts on human health remain underexplored. In this study, we address this gap by providing the first spatially explicit assessment of zoonotic risks associated with invasive mammals in Europe. Aligning with a One Health–One Biosecurity framework [23], our results support the idea that preventing and managing biological invasions can help reduce public health risks while also protecting biodiversity and ecosystem functioning.

We focused on eight invasive mammals from the EU Union List, all known or suspected reservoirs of zoonotic pathogens [35]. These species were introduced to Europe over the past 150 years, mostly through intentional releases followed by escape from confinement, a common introduction pathway for large mammals [39,42]. They have since spread widely across the continent.

Our literature review showed that the selected invasive mammals are associated with a diverse range of pathogens, supporting the idea that invasive hosts can play a role in introducing and increasing pathogens in areas they invade. It also highlights the importance of considering IAS when assessing zoonotic risks. In addition, many pathogens are shared among multiple hosts, increasing the risk of zoonotic spillover.

The public health impact of IAS is not merely a function of the number of pathogens they host, but also the severity of those pathogens. This is important for prioritizing surveillance and management, because IAS associated with fewer pathogens may still represent a disproportionate threat if they host high-risk pathogens. For example, *T. sibiricus*, though linked to only three pathogens, carries *Borrelia burgdorferi* sensu lato (BSL-3). In contrast, *N. procyonoides* hosts 32 pathogens, most of lower severity (BSL-1 or BSL-2), showing that a higher pathogen count does not automatically imply a high public health risk. While Echinococcosis (1122 cases) and Leptospirosis (1544 cases) have the highest incidence in Europe, Lyme neuroborreliosis is particularly concerning despite having fewer reported cases (317), due to its high prevalence in specific invasive hosts like *T. sibiricus* (35 %). This shows the importance of considering both incidence and host-specific prevalence when prioritizing surveillance.

To evaluate current and future risks, and assuming IAS can expand into all climatically suitable areas, we modeled the potential distribution of the selected invasive mammals under current and 2050 climate scenarios. Unlike previous SDMs studies primarily focused on biodiversity [17,18], this is the first to apply them specifically to human health impacts, providing a spatial tool for anticipating where invasive hosts may increase opportunities for zoonotic transmission.

Our results show that the current distribution of invasive mammals is primarily determined by accessibility to urban centers together with temperature variables, especially annual mean temperature (bio1) and temperature seasonality (bio4), in line with previous findings [32].

Climate change is expected to shift the mammals' ranges northward, making Southern Europe less suitable for their establishment and Central and Northern Europe more favorable, due to milder winters and increased precipitation [8,17,32,39]. From a public health perspective, this suggests that areas currently considered at moderate risk may become increasingly relevant for surveillance as invasive mammals expand due to climate change.

Species responses to climate change were highly variable, with *T. sibiricus* projected to lose up to 45 % of its suitable area and *S. carolinensis* to gain up to 26 % under the high-emission scenario. These findings are consistent with global projections [9], and suggest that future zoonotic risk will not increase uniformly across invasive mammals and reinforce the need to interpret risk through species-specific ecology rather than broad generalisations.

However, many established species such as *P. lotor*, *M. coypus*, and *O. zibethicus* have already realized much of their potential range due to early introductions and long residence times [46], and they are expected to persist in established regions due to high adaptability and reproductive rates [38]. Nonetheless, our projections indicate that expansion remains possible in currently unoccupied but climatically suitable areas, especially in Western and Southwestern Europe, meaning that management and monitoring should not assume that these invasions are fully saturated.

Other species are in earlier stages of invasion. *S. carolinensis* and *T. sibiricus* are expected to expand, particularly in France and Germany. The Javan mongoose (*H. javanicus*), despite being introduced in the early 20th century, remains rare in Europe due to limited habitat suitability. The ring-tailed coati (*N. nasua*), more recently introduced in 2003, currently has few records but may expand in regions surrounding the British Channel (including northern France, southern England, Belgium, and nearby coastal regions). It exemplifies the concept of invasion debt, where species remain at low population levels before potentially spreading rapidly [16,33], highlighting the importance of early detection and monitoring to prevent zoonotic risks.

A novel contribution of this study is the development of disease hotspot maps that identify where the potential for zoonotic transmission from invasive mammals is likely to be highest under current conditions. These hotspot areas align with major trade routes and areas of intense human activity (including the British Channel region, France, Germany, and the Benelux region), where invasive mammals can easily spread [17,19]. This spatial pattern helps identify priority regions for targeted surveillance at the interface between IAS management and public health.

Our analysis also explored the degree of potential human exposure to the selected invasive mammals, which ranged widely among species: from 23 % for *H. javanicus* to 99 % for *M. coypus* (Table 5). The high exposure associated with *M. coypus* (99 %) reflects its ecological versatility and ability to thrive across diverse climates and landscapes, making it a widespread and persistent invader in Europe [36]. Human exposure should be considered an important complementary criterion when prioritizing invasive mammals for surveillance and management. At the same time, these human exposure estimates should be interpreted as indicators of potential contact rather than confirmed interaction, as they are based on spatial overlap between habitat suitability and population density.

## 5 Limitations

This study represents an initial step in understanding the role of invasive mammals in zoonotic disease transmission, but has several limitations. First, the eight species analyzed are management priorities under the EU Union List, but this does not necessarily mean they are the most important invasive mammals for zoonotic risk across Europe. Second, although the predictors used capture broad-scale suitability patterns, model performance could likely be improved by including additional variables related to propagule pressure, dispersal and establishment, as well as finer habitat descriptors. However, such predictors are not generally available at the global extent and 10 × 10 km resolution applied here. Third, species occurrence data may be affected by sampling bias, with a higher representation of accessible areas. In addition, model performance was assessed using random cross-validation, which is widely used but may underestimate prediction error compared with spatially structured approaches such as block cross-validation [4,43].

## 6 Conclusions

Invasive alien mammals pose risks not only to biodiversity and ecosystem functioning but also to human health, as many of these species act as reservoirs of zoonotic pathogens. From a One Health perspective, our study provides the first spatially explicit assessment of zoonotic risks associated with invasive mammals in Europe and offers a useful foundation for future work as data and modeling approaches continue to improve.

By combining species distribution models with pathogen prevalence data, we identified current disease hotspot areas where the potential for zoonotic transmission is highest, particularly across Western and Central Europe.

Overall, our findings show that managing invasive mammals can contribute to both ecosystem protection and public health. The framework presented here can help guide surveillance, prevention, and control strategies aimed at mitigating the threats posed by emerging zoonotic diseases.

## CRediT authorship contribution statement

**Paola Monguilod:** Writing – original draft, Methodology, Investigation, Conceptualization. **Belinda Gallardo:** Writing – review & editing, Validation, Supervision, Resources, Project administration, Methodology, Investigation, Funding acquisition, Conceptualization.

## Funding

This study has received funding from the 10.13039/501100003339Spanish National Research Council (CSIC), through its program JAE-Intro 2022, as part of the training plan JAEINT22_EX_0788 and from the Association for the Integration of Ecosystem Services (AISECO).

## Declaration of competing interest

The authors declare no conflicts of interest.

## Data Availability

The species occurrence data used in this study were sourced from the Global Biodiversity Information Facility database (GBIF; https://www.gbif.org), the European Alien Species Information Network (EASIN; https://easin.jrc.ec.europa.eu/easin, and various original literature compilations [5,17]. The data regarding the cases of the zoonotic diseases studied are available from The European Surveillance System (TESSy) repository (https://www.ecdc.europa.eu/en/publications-data/european-surveillance-system-tessy). Restrictions apply to the availability of these data, which were used under license for this study.

## Appendix A Supplementary data

Supplementary material Image 1
