# Supplementary material for: Invasive alien mammals pose zoonotic risks to human health in Europe
Source: One Health. 2025 Dec 19;22:101307. doi: 10.1016/j.onehlt.2025.101307 (PMC12811535; doi:10.1016/j.onehlt.2025.101307)
Supplement: Supplementary file 1 — Supplementary material [file mmc1.docx]

Supplementary information for:

Invasive alien mammals pose zoonotic risks to human health in Europe

This file contains:

- Tables S1 to S15
- Figures S1 to S7
- List of references

**Table S1**: Summary of the data extracted during the literature review, featuring the pathogens associated with each IAS, along with their prevalence. The criteria for inclusion/exclusion of research articles are described in the text in the methods section. Literature review conducted in March 2023. Sample size: number of individuals analyzed in the study to calculate prevalence. Reliability of source: 1=High, 2=Medium and 3=Low. The severity is displayed only for sources with reliability 1 and 2. Biosecurity level (BSL) of laboratories handling specific diseases, as an indicator of severity. It ranges from BSL-1 to BSL-4. See more details in Methods. More information about the IAS (taxonomy, origin, pathway, date of first introduction in Europe) can be found in Table 1.

| **Invasive Alien Species** | **Pathogen** | **Sample size** | **Prevalence (%)** | **Severity (BSL)** | **Reliability of source** | **Country** | **Reference** |
| --- | --- | --- | --- | --- | --- | --- | --- |
| ***Herpestes javanicus***  (É. Geoffroy Saint-Hilaire, 1818) | *Salmonella* spp. | 48 | 13-55% | 2 | 1 | Barbados | ^1^ |
|  | Thermophilic *Campylobacter* spp. | 48 | 9.09% | 2 | 1 | Barbados | ^1^ |
|  | *Hepatitis E virus* | 84 | 8.3% | 3 | 1 | Japan | ^2^ |
|  | *Japanese encephalitis virus* (JEV) | 240 | 35.4% | 3 | 1 | Japan | ^3^ |
|  | *Leptospira* | 148 | 8.1% | 3 | 1 | The Caribbean | ^4^ |
|  | *Hepatitis E virus* | 100 | 21% | 3 | 1 | Japan | ^5^ |
| ***Myocastor coypus***  (Molina, 1782) | *Blastocystis* | 308 | 14.3% | 1 | 1 | China | ^6^ |
|  | Carbapenem-Resistant and Zoonotic *Aeromonas* spp. | 26 | 53.8% | 1 | 1 | South Korea | ^7^ |
|  | *Leptospira* | 176 | 38.0% | 3 | 1 | Argentina | ^8^ |
|  | *Toxoplasma gondii* | 176 | 27.8 % | 2 | 1 | Argentina | ^8^ |
|  | *Chlamydophila psittaci* | 176 | 21.0% | 2 | 1 | Argentina | ^8^ |
|  | *Streptococcus equi* subspecies zooepidemicus, | 176 | 15.9 % | 2 | 1 | Argentina | ^8^ |
|  | *Encephalomyocarditis virus* | 176 | 3.4 % | 2 | 1 | Argentina | ^8^ |
|  | *Giardia duodenalis* | 308 | 12.3% | 2 | 1 | China | ^9^ |
|  | *Fasciola hepatica* | 1 | 100%, | - | 3 | Uruguay | ^10^ |
|  | *Enterocytozoon bieneusi* | 308 | 41.2% | 2 | 1 | China | ^11^ |
|  | *Strongyloides myopotami* | 153 | 63.40% | 1 | 1 | Italy | ^12^ |
|  | *Trichostrongylus duretteae* | 153 | 28.10% | 1 | 1 | Italy | ^12^ |
|  | *Eimeria coypi* | 153 | 86.30% | 1 | 1 | Italy | ^12^ |
|  | *Eimeria seideli* | 153 | 6.80% | 1 | 1 | Italy | ^12^ |
|  | *Aeromonas rivipollensis* KN-Mc-11N1 | 1 | 100% | - | 3 | South Korea | ^13^ |
|  | *Toxoplasma gondii* | 74 | 59.4% | 2 | 1 | Italy | ^14^ |
|  | *Aeromonas* spp. (*A. hydrophila*, *A. caviae*, and *A. dhakensis*) | - | - | - | 3 | South Korea | ^7^ |
|  | *Echinococcus multilocularis* | 231 | 0.4% | 3 | 1 | France | ^15^ |
|  | *Taenia* sp. | 531 | 1.3% | 3 | 1 | France | ^15^ |
|  | *Strongyloides myopotami* | 86 | 99% | 1 | 1 | Japan | ^16^ |
| ***Nasua nasua*** (Linnaeus, 1766) | Filarial nematodes | 75 | 81.6% | 2 | 1 | Brazil | ^17^ |
|  | *Clostridium difficile* | 46 | 6.5% | 2 | 1 | Brazil | ^18^ |
|  | *Dirofilaria incrassata* | 2 | 100% | - | 3 | Brazil | ^19^ |
|  | *Trypanosoma cruzi* | - | 53.5% | 3 | 2 | Brazil | ^20^ |
|  | *Trypanosoma evansi* | - | 42.0% | 3 | 2 | Brazil | ^20^ |
|  | Hemotropic *Mycoplasma* sp. | 49 | 85.7% | 3 | 1 | Brazil | ^21^ |
|  | *Bartonella machadoae* | 49 | 24.5% | 3 | 1 | Brazil | ^21^ |
|  | *Anaplasma* sp. | 49 | 14.3% | 3 | 1 | Brazil | ^21^ |
|  | *Hepatozoon procyonis* | 49 | 6% | 2 | 1 | Brazil | ^21^ |
|  | Hemotropic *Mycoplasma* sp. | 2 | 100% | - | 3 | Brazil | ^22^ |
|  | *Angiostrongylus minasensis* n. sp. | 10 | 100% | 2 | 2 | Brazil | ^23^ |
|  | *Babesia* spp. | - | - | - | 3 | Brazil | ^24^ |
|  | *Rickettsia* spp. | - | - | - | 3 | Brazil | ^24^ |
|  | *Trypanosoma cruzi* | 20 | 35% | 3 | 2 | Costa Rica | ^25^ |
|  | *Coccidia* | 3 | 66.66% | 3 | 2 | Norway | ^26^ |
| ***Nyctereutes procyonoides*** (Gray, 1834) | *Toxoplasma gondii* | 292 | 42.7% | 2 | 1 | Denmark | ^27^ |
|  | *Alaria alata* | 292 | 32.9% | 2 | 1 | Denmark | ^27^ |
|  | *Alaria alata* | 10 | 30% | 2 | 2 | Austria | ^28^ |
|  | *Echinococcus multilocularis* | 10 | 10% | 3 | 2 | Austria | ^28^ |
|  | *Uncinaria stenocephala* | 10 | 40% | 2 | 2 | Austria | ^28^ |
|  | *Mesocestoides* spp | 10 | 40% | 2 | 2 | Austria | ^28^ |
|  | *Molineus* spp. | 10 | 30% | 2 | 2 | Austria | ^28^ |
|  | *Toxocara canis* | 10 | 20% | 2 | 2 | Austria | ^28^ |
|  | *Taenia* spp. | 10 | 20% | 3 | 2 | Austria | ^28^ |
|  | *Isthmiophora melis* | 10 | 20% | 2 | 2 | Austria | ^28^ |
|  | *Dipylidium caninum* | 10 | 10% | 2 | 2 | Austria | ^28^ |
|  | *Toxascaris leonina* | 10 | 10% | 2 | 2 | Austria | ^28^ |
|  | *Echinococcus multilocularis* | - | - | 3 | 1 | Germany | ^29^ |
|  | *Enterocytozoon bieneusi* | 162 | 10.5 % | 2 | 1 | China | ^30^ |
|  | *Enterocytozoon bieneusi* | 49 | 4.1% | 2 | 1 | China | ^31^ |
|  | *Alaria alata* | 53 | 94.3% | 2 | 1 | Poland | ^32^ |
|  | *Apophallus* spp. | 53 | 15.10% | 2 | 1 | Poland | ^32^ |
|  | *Mesocestoides* spp. | 53 | 24.50% | 2 | 1 | Poland | ^32^ |
|  | Toxascaris nematodes | 53 | 15.10% | 2 | 1 | Poland | ^32^ |
|  | *Echinostomatidae* | 53 | 18.90% | 2 | 1 | Poland | ^32^ |
|  | *Molineus* spp. | 53 | 41.50% | 2 | 1 | Poland | ^32^ |
|  | *Capillaria* spp. | 53 | 20% | 2 | 1 | Poland | ^32^ |
|  | *Taenia spp.* | 53 | 1.90% | 3 | 1 | Poland | ^32^ |
|  | *Cryptosporidium* spp. | 162 | 10.5% | 2 | 1 | China | ^33^ |
|  | *Alaria alata* | 99 | 96.50% | 2 | 1 | Lithuania | ^34^ |
|  | *Trichinella* spp. | 99 | 29.30% | 2 | 1 | Lithuania | ^34^ |
|  | *Eucoleus aerophilus* | 99 | 30.20% | 2 | 1 | Lithuania | ^34^ |
|  | *Crenosoma vulpis* | 99 | 15.10% | 2 | 1 | Lithuania | ^34^ |
|  | *Capillaria plica* | 99 | 11.30% | 2 | 1 | Lithuania | ^34^ |
|  | *Ctenocephalides putorii* | 99 | 51.50% | 1 | 1 | Lithuania | ^34^ |
|  | *Toxocara canis* | 99 | 17.60% | 2 | 1 | Lithuania | ^34^ |
|  | *Uncinaria stenocephala* | 99 | 98.80% | 2 | 1 | Lithuania | ^34^ |
|  | *Pygidiopsis summa* | 99 | 3.00% | 2 | 1 | Denmark | ^35^ |
|  | *Cryptocotyle* spp. | 99 | 15.20% | 2 | 1 | Denmark | ^35^ |
|  | *Toxocara canis* | 99 | 13.10% | 2 | 1 | Denmark | ^35^ |
|  | *Uncinaria stenocephala* | 99 | 48.50% | 2 | 1 | Denmark | ^35^ |
|  | *Mesocestoides* spp. | 99 | 23.20% | 2 | 1 | Denmark | ^35^ |
|  | *Taenia* spp. | 99 | 2.00% | 3 | 1 | Denmark | ^35^ |
|  | *Dipylidium caninum* | 99 | 5.10% | 2 | 1 | Denmark | ^35^ |
|  | *Mesorchis denticulatus* | 99 | 38.40% | 2 | 1 | Denmark | ^35^ |
|  | *Alaria alata* | 99 | 69.70% | 2 | 1 | Denmark | ^35^ |
|  | *Uncinaria stenocephala* | 225 | 97.6% | 2 | 1 | Estonia | ^36^ |
|  | *Alaria alata* | 225 | 68.3% | 2 | 1 | Estonia | ^36^ |
|  | *Anaplasma phagocytophilum* | 122 | 23% | 3 | 1 | Germany | ^37^ |
|  | *Echinococcus multilocularis* | 1252 | 12.0% | 3 | 2 | Germany | ^38^ |
|  | *Echinococcus multilocularis* | 249 | 1.6 | 3 | 1 | Estonia | ^39^ |
|  | *Francisella tularensis* | 345 | 12.80% | 4 | 1 | Germany | ^40^ |
|  | *Rabies virus* | 2277 | 21% | 4 | 1 | Lithuania | ^41^ |
|  | *Trichinella britovi* | 45 | 39.82% | 2 | 1 | Poland | ^42^ |
|  | *Trichinella* spp. | 1527 | 1.90% | 2 | 1 | Germany | ^43^ |
|  | *Rabies virus* | - | 28.9% | 4 | 2 | Lithuania | ^44^ |
| ***Procyon lotor*** (Linnaeus, 1758) | *Baylisascaris procyonis* | - | 71% | 2 | 2 | Austria | ^28^ |
|  | *Molineus* spp. | 8 | - | - | 3 | Austria | ^28^ |
|  | *Baylisascaris procyonis* | 21 | 33.3% | 2 | 1 | Italy | ^45^ |
|  | *Cryptosporidium* | 30 | 13.3% | 2 | 1 | Iran | ^46^ |
|  | *Giardia duodenalis* | 66 | 27% | 2 | 1 | Germany and Luxembourg | ^47^ |
|  | *Moellerella wisconsensis* | 1 | - | - | 3 | - | ^48^ |
|  | *Blastocystis* | 30 | 16.66% | 1 | 1 | Iran | ^49^ |
|  | *Baylisascaris procyonis* | 32 | 75% | 2 | 1 | Germany | ^50^ |
|  | *Leptospira* | 65 | 30% | 3 | 1 | United States | ^51^ |
|  | *Canine distemper virus*, *Hepatitis E virus*, *Cryptosporidium*, *Neospora, Sarcocystis*, *Toxoplasma*, *Alaria, Baylisascaris*, *Mesocestoides*, *Sarcoptes* | - | - | - | 3 | Germany | ^52^ |
|  | *Baylisascaris procyonis* | 18 | 0.11 | 2 | 2 | Denmark | ^53^ |
|  | *Baylisascaris procyonis* | - | - | - | 3 | Germany | ^54^ |
|  | *Baylisascaris procyonis* | 12 | 0.25 | 2 | 2 | Japan | ^55^ |
|  | *Borrelia afzelii* | 559 | 0.2% | 2 | 1 | Japan | ^56^ |
|  | *Borrelia garinii* | 559 | 0.2% | 2 | 1 | Japan | ^56^ |
|  | *Francisella tularensis* | 559 | 0.5% | 4 | 1 | Japan | ^56^ |
|  | *Orientia tsutsugamushi* (Gilliam type) | 559 | 1.4% | 3 | 1 | Japan | ^56^ |
|  | *Orientia tsutsugamushi* (Kuroki type majority) | 193 | 16.1% | 3 | 1 | Japan | ^56^ |
|  | *Rickettsia japonica* | 193 | 7.3% | 3 | 1 | Japan | ^56^ |
|  | *Capillaria* sp. | 55 | 25.5% | 2 | 1 | Poland | ^57^ |
|  | *Echinostomatidae* | 55 | 34.5 | 2 | 1 | Poland | ^57^ |
|  | *Cryptosporidium* sp. | 49 | 34.7% | 2 | 1 | Germany and Poland | ^58^ |
|  | *Enterocytozoon bieneusi* | 49 | 4.1% | 2 | 1 | Germany and Poland | ^58^ |
|  | *Sarcocystis kirkpatricki* | 12 | 8.3%. | 2 | 2 | Germany | ^59^ |
|  | *Strongyloides procyonis* | 233 | 28.3% | 2 | 1 | Japan | ^60^ |
|  | *Toxascaris* sp. | 4 | 50% | - | 3 | Norway | ^61^ |
|  | *Toxocara* sp. | 4 | 50% | - | 3 | Norway | ^61^ |
|  | *Baylisascaris procyonis* | 4 | 100% | - | 3 | Norway | ^61^ |
|  | Salmonella spp. | 459 | 5.7% | 2 | 1 | Japan | ^62^ |
|  | *Yersinia* | 459 | 38,60% | 2 | 1 | Japan | ^62^ |
|  | *Campylobacter* spp. | 459 | 1,30% | 2 | 1 | Japan | ^62^ |
|  | *Salmonella* spp. (*Salmonella enterica*) | 70 | *Salmonella* spp.: 5.7 (*Salmonella enterica*: 2.85%) | 2(2) | 1 | Poland | ^63^ |
|  | *Yersinia* spp. | 70 | 4.28% | 2 | 1 | Poland | ^63^ |
|  | *Straphylococcus* coagulase-positive strains | 70 | 37.51% | 2 | 1 | Poland | ^63^ |
|  | *Listeria* spp. | 70 | 7.14% | 2 | 1 | Poland | ^63^ |
| ***Sciurus carolinensis*** (Gmelin, 1788) | *MCMV virus* | 4 | 100% | - | 3 | Wales | ^64^ |
|  | *Ectromelia virus* | 19 | 5.30% | 3 | 2 | Wales | ^64^ |
|  | *MAV FL and K87 virus* | 15 | 60% | 2 | 2 | Wales | ^64^ |
|  | *MVM virus* | 15 | 20% | 2 | 2 | Wales | ^64^ |
|  | *Reovirus 3 virus* | 15 | 80% | 2 | 2 | Wales | ^64^ |
|  | *Rotavirus virus* | 8 | 62.50% | - | 3 | Wales | ^64^ |
|  | *Coronavirus* | 19 | 36.80% | 2 | 2 | Wales | ^64^ |
|  | *Sendai virus* | 19 | 52.60% | 2 | 2 | Wales | ^64^ |
|  | *PVM virus* | 15 | 20% | 2 | 2 | Wales | ^64^ |
|  | *LCMV virus* | 19 | 21% | 2 | 2 | Wales | ^64^ |
|  | *GDVII virus* | 15 | 13.30% | 2 | 2 | Wales | ^64^ |
|  | *Mycoplasma pulmonis* | 15 | 26.7% | 3 | 2 | Wales | ^64^ |
|  | *Bartonella vinsonii* | 20 UK, 18 US | 20% UK, 28% US | 3 | 2 | England and United States | ^65^ |
|  | *Sendai virus*, *Lymphocytic choriomeningitis virus*, *Borrelia burgdorferi* | - | - | - | 3 | Europe | ^66^ |
|  | *Borrelia lusitaniae*, *Coxiella burnetii* | - | - | - | 3 | Italy | ^67^ |
|  | *Borrelia burgdorferi Sensu Lato (Lyme borreliosis)* | 679 | 11.9% | 3 | 1 | Scotland | ^68^ |
|  | *Tick-borne encephalitis virus* (TBEV) | 158 | 1.9-2.5% | 3 | 1 | Italy | ^69^ |
|  | *Usutu virus* (USUV) | 158 | 3.2-3.8% | 3 | 1 | Italy | ^69^ |
|  | *West Nile virus* (WNV) | 158 | 0.6% | 3 | 1 | Italy | ^69^ |
|  | *Variegated squirrel bornavirus 1* (VSBV-1) | 328 | 5% | 2 | 1 | Germany | ^70^ |
| ***Tamias sibiricus*** (Laxmann, 1769) | *Anaplasma phagocytophilum* | - | - | - | 3 | Siberia | ^71^ |
|  | *Borrelia burgdorferi* Sensu Lato (Lyme disease) | 335 | 35.2% | 3 | 1 | France | ^72^ |
|  | *Borrelia burgdorferi* Sensu Lato (Lyme disease) | - | 10% | 3 | 2 | France | ^72^ |
|  | *Borrelia afzelii* | - | 7% | - | 3 | France | ^72^ |
|  | *Borrelia burgdorferi* | - | 33.33 | 3 | 2 | France | ^73^ |
| ***Ondatra zibethicus*** (Linnaeus, 1766) | *Hydatigera taeniaeformis* | 130 | 48.9% | 2 | 1 | Germany | ^74^ |
|  | *Taenia polyacantha* | 130 | 13% | 3 | 1 | Germany | ^74^ |
|  | *Bartonella* | 1 | 100% | - | 3 | Belgium | ^75^ |
|  | *Francisella tularensis* | 12 | 33% | 4 | 2 | Pennsylvania | ^76^ |
|  | *Clostridium piliforme*  (Tyzzer's disease) | 12 | 33% | 2 | 2 | Pennsylvania | ^76^ |
|  | *Versteria* sp | 1 | 100% | - | 3 | Pennsylvania | ^77^ |
|  | *Bordetella bronchiseptica* | 3 | 33% | - | 3 | North America | ^78^ |
|  | *Campylobacter jejuni* | 189 | 48% | 2 | 1 | North America | ^78^ |
|  | *Chlamydia psittaci* | 2 | 100% | - | 3 | North America | ^78^ |
|  | *Citrobacter freundii* | 3 | 67% | - | 3 | North America | ^78^ |
|  | *Clostridium piliforme* (Tyzzer's disease) | 18 | 100% | 2 | 2 | North America | ^78^ |
|  | *Francisella philomiragia* | 1 | 100% | - | 3 | North America | ^78^ |
|  | *Francisella tularensis* | 6 | 100% | - | 3 | North America | ^78^ |
|  | *Providencia stuartii* | 3 | 33.33% | - | 3 | North America | ^78^ |
|  | *Pseudomonas aeruginosa* | 3 | 33.33% | - | 3 | North America | ^78^ |
|  | *Staphylococcus* sp. | 1 | 100% | - | 3 | North America | ^78^ |
|  | *Yersinia ruckeri* | 1 | 100% | - | 3 | North America | ^78^ |
|  | *Cryptosporidium* sp. | 44 | 50% | 2 | 1 | North America | ^78^ |
|  | *Giardia duodenalis* | 23 | 78.26% | 2 | 1 | North America | ^78^ |
|  | *Giardia* sp. | 44 | 65.91% | 2 | 1 | North America | ^78^ |
|  | *Toxoplasma gondii* | 30 | 60% | 2 | 1 | North America | ^78^ |
|  | *Trichomonas* sp. | 25 | 100% | 2 | 1 | North America | ^78^ |
|  | *Echinochasmus schwartzi* | 36 | 58.33% | 2 | 1 | North America | ^78^ |
|  | *Echinostoma revolutum* | 81, 35 | 92.59%, 51.43% | 2 | 1 | North America | ^78^ |
|  | *Echinostomum* sp. | 63 | 84.13% | 2 | 1 | North America | ^78^ |
|  | *Hymenolepis evaginata* | 114 | 58.77% | 2 | 1 | North America | ^78^ |
|  | *Hymenolepis* sp. | 205, 34 | 30.73%, 11.76% | 2 | 1 | North America | ^78^ |
|  | *Calodium hepatica* | 360 | 61.39% | 2 | 1 | North America | ^78^ |
|  | *Giardia* | 1 | 100% | - | 3 | United States | ^79^ |
|  | *Echinococcus multilocularis* | 1718 | 11.18% | 3 | 1 | Belgium | ^80^ |
|  | *Echinococcus*  *multilocularis* | 991 | 4.1% | 3 | 1 | Germany | ^81^ |
|  | *Taenia taeniaeformis* | 991 | 42.3% | 3 | 1 | Germany | ^81^ |
|  | *Taenia crassiceps* | 991 | 2.7% | 3 | 1 | Germany | ^81^ |
|  | *Taenia polyacantha* | 991 | 0.4% | 3 | 1 | Germany | ^81^ |
|  | *Taenia martis* | 991 | 3.4% | 3 | 1 | Germany | ^81^ |
|  | *Anoplocephalidae* | 991 | 3.4% | 2 | 1 | Germany | ^81^ |
|  | *Giardia* spp. | 234 | 75.2% | 2 | 1 | Germany | ^82^ |
|  | *Hantavirus* | 197 | 8% | 3 | 1 | Germany | ^83^ |
|  | *Leptospira* | - | - | - | 3 | France | ^84^ |
|  | *Echinococcus multilocularis* | 285 | 0.70% | 3 | 1 | France | ^15^ |
|  | *Taenia* sp. | 285 | 31.80% | 3 | 1 | France | ^15^ |

**Table S2**. Variables included in the species distribution models as potential predictors.

| **Type** | **Variable** | **Extent** | **Description** | **Resolution** | **References** |
| --- | --- | --- | --- | --- | --- |
| Anthropogenic | Accessibility | Global | Time of travel required to arrive to the closest urban center (<50.000 inhabitants), integrating both distance to urban areas and the presence of transportation networks (days^-1^) | 10x10 km | (Weiss et al., 2018) |
| Environmental | Elevation | Global | Height or altitude of a specific location above sea level (m) | 10x10 km | (Amante & Eakins, 2009) |
| Climatic | Bio1 | Global | Annual Mean Temperature (^o^C) | 10x10 km | (Karger & Zimmermann, 2019) |
|  | Bio3 | Global | Isothermality (dimensionless) | 10x10 km | (Karger & Zimmermann, 2019) |
|  | Bio4 | Global | Temperature Seasonality  (dimensionless) | 10x10 km | (Karger & Zimmermann, 2019) |
|  | Bio5 | Global | Maximum Temperature of Warmest Month  (^o^C) | 10x10 km | (Karger & Zimmermann, 2019) |
|  | Bio15 | Global | Precipitation Seasonality  (mm) | 10x10 km | (Karger & Zimmermann, 2019) |
|  | Bio18 | Global | Precipitation of Warmest Quarter  (mm) | 10x10 km | (Karger & Zimmermann, 2019) |
|  | Bio19 | Global | Precipitation of Coldest Quarter  (mm) | 10x10 km | (Karger & Zimmermann, 2019) |

**REFERENCES:**

Amante, C., & Eakins, B. W. (2009). ETOPO1 arc-minute global relief model: procedures, data sources and analysis. https://repository.library.noaa.gov/view/noaa/1163

Karger, D. N., & Zimmermann, N. E. (2019). Climatologies at high resolution for the earth land surface areas CHELSA V1. 2: Technical specification. Swiss Federal Research Institute WSL, Switzerland. https://doi.org/10.5061/dryad.kd1d4

Weiss, D. J., Nelson, A., Gibson, H. S., Temperley, W., Peedell, S., Lieber, A., Hancher, M., Poyart, E., Belchior, S., Fullman, N., Mappin, B., Dalrymple, U., Rozier, J., Lucas, T. C. D., Howes, R. E., Tusting, L. S., Kang, S. Y., Cameron, E., Bisanzio, D., … Gething, P. W. (2018). A global map of travel time to cities to assess inequalities in accessibility in 2015. Nature, 553(7688), 333–336. https://doi.org/10.1038/nature25181

**Table S3**. Description of the three different 2050 future scenarios considered in the study (CHELSA <https://chelsa-climate.org/>, 2023).

These scenarios integrate various greenhouse gas Representative Concentration Pathways (RCPs, Van Vuuren et al., 2011) with socio-economic Shared Socioeconomic Pathways (SSPs, Riahi et al., 2017), as detailed in the IPCC 6th Assessment Report (<https://www.ipcc.ch/assessment-report/ar6/>).

| **Scenario** | **Description** |
| --- | --- |
| **ssp126** | SSP1-RCP 2.6: A Sustainability Path (Low emissions scenario) |
|  | Moderate climate impacts as societies transition towards sustainability. Low challenges in mitigation and adaptation. Emphasis on inclusive development, education, and health. Economic growth is aligned with well-being. Reduced greenhouse gas emissions and manageable climate consequences.  This scenario represents an increase of 0.92ºC in maximum Bio1 across Europe. |
| **ssp370** | SSP3-RCP 7: A Regional Rivalry Path (Business as usual, BAU, scenario) |
|  | Severe climate impacts due to global cooperation challenges. Fragmented world with a focus on domestic or regional concerns over climate issues. Decline in education and technology investments, slow economic growth, and worsening inequality, leading to increased vulnerability to extreme weather events.  This scenario represents an increase of 1.57ºC in maximum Bio1 across Europe. |
| **ssp585** | SSP5-RCP 8.5: A Fossil-fueled Development Path (High emissions scenario) |
|  | Significant climate impacts despite low adaptation challenges. Rapid economic growth driven by fossil fuels increases greenhouse gas emissions. Technological advancements may help locally but worsen global climate issues. Uncertainty about the effectiveness of geo-engineering and technological solutions to prevent extreme climate events.  This scenario represents an increase of 1.82ºC in maximum Bio1 across Europe. |

**NOTES:**

Riahi, K. *et al.* (2017) ‘The Shared Socioeconomic Pathways and their energy, land use, and greenhouse gas emissions implications: An overview’, *Global Environmental Change*, 42, pp. 153–168. Available at: https://doi.org/10.1016/J.GLOENVCHA.2016.05.009.

Van Vuuren, D.P. *et al.* (2011) ‘The representative concentration pathways: An overview’, *Climatic Change*, 109, pp. 5–31. Available at: https://doi.org/10.1007/S10584-011-0148-Z/TABLES/4.

**Table S4**. Overview of IAS occurrences utilized in Species Distribution Models, including scientific names, identification codes (ID), DOI references, and occurrence data from various sources (see Table footnotes). The table presents total occurrences and gridded occurrences (one occurrence per 10x10km) for each species. NA= not available.

| **Scientific name** | **ID** | **GBIF** | **DOI** | **Gallardo et al. (2017)** | **EASIN (2017)** | **EASIN (2017)** | **EASIN (2019)** | **EASIN (2019) updates** | **EASIN total** | **Ascensao et al. (2021)** | **TOTAL OCCURRENCES** | **GRIDDED OCCURRENCES** |
| --- | --- | --- | --- | --- | --- | --- | --- | --- | --- | --- | --- | --- |
| *Herpestes javanicus* | Hjava | 7901 | 10.15468/dl.f9gpws | NA | 40 | NA | NA | NA | 40 | NA | 7941 | 310 |
| *Myocastor coypus* | Mcoyp | 56336 | 10.15468/dl.svbygj | 7050 | 3366 | NA | NA | 2723 | 6089 | 41 | 69516 | 5277 |
| *Nasua nasua* | Nnasu | 2498 | 10.15468/dl.e2wqth | NA | 9 | NA | NA | NA | 9 | NA | 2507 | 618 |
| *Nyctereutes procyonoides* | Nproc | 23982 | 10.15468/dl.vk2qa3 | 1772 | NA | NA | 5880 | NA | 5880 | NA | 31634 | 2563 |
| *Ondatra zibethicus* | Ozibe | 557413 | 10.15468/dl.484ad2 | 3152 | NA | 6155 | NA | NA | 6155 | 7 | 566727 | 8732 |
| *Procyon lotor* | Ploto | 50465 | 10.15468/dl.gzuftb | 8420 | 6636 | NA | NA | 6712 | 13348 | 20 | 72253 | 10788 |
| *Sciurus carolinensis* | Scaro | 227473 | 10.15468/dl.t7wryw | 5166 | 5301 | NA | NA | 5253 | 10554 | NA | 243193 | 6263 |
| *Tamias sibiricus* | Tsibi | 11844 | 10.15468/dl.n4yaq5 | 636 | 146 | NA | NA | 112 | 258 | NA | 12738 | 980 |
|  |  |  |  |  |  |  |  |  |  | TOTAL | 1006509 | 35531 |

**NOTES:**

Ascensáo, F., D’Amico, M., Martins, R. C., Rebelo, R., Barbosa, A. M., Bencatel, J., Barrientos, R., Abellán, P., Tella, J. L., Cardador, L., Anadón, J. D., Carrete, M., Murgui, E., Fernandes, P., Santos, S. M., Mira, A., da Luz Mathias, M., Tiago, P., Casabella, E., … Capinha, C. (2021). Distribution of alien tetrapods in the Iberian Peninsula. NeoBiota, 64, 1–21. <https://doi.org/10.3897/NEOBIOTA.64.55597>.

Gallardo, B., Aldridge, D. C., González-Moreno, P., Pergl, J., Pizarro, M., Pyšek, P., Thuiller, W., Yesson, C., & Vilà, M. (2017). Protected areas offer refuge from invasive species spreading under climate change. Global Change Biology, 23(12), 5331–5343. <https://doi.org/10.1111/GCB.13798>.

GBIF database (<https://www.gbif.org>).

EASIN database (<https://easin.jrc.ec.europa.eu/easin>).

**Table S5.** True Skill Statistic (TSS) values for the calibration of the ensemble models.

| **Species** | **Calibration metric** |
| --- | --- |
| *Herpestes javanicus* | 0.784 |
| *Myocastor coypus* | 0.780 |
| *Nasua nasua* | 0.752 |
| *Nyctereutes procyonoides* | 0.854 |
| *Ondatra zibethicus* | 0.848 |
| *Procyon lotor* | 0.909 |
| *Sciurus carolinensis* | 0.842 |
| *Tamias sibiricus* | 0.712 |

|  | **GLM** | | | | **GBM** | | | | **RF** | | | | **GAM** | | | |
| --- | --- | --- | --- | --- | --- | --- | --- | --- | --- | --- | --- | --- | --- | --- | --- | --- |
| **Species** | **Cutoff** | **Sensitivity** | **Specificity** | **Validation** | **Cutoff** | **Sensitivity** | **Specificity** | **Validation** | **Cutoff** | **Sensitivity** | **Specificity** | **Validation** | **Cutoff** | **Sensitivity** | **Specificity** | **Validation** |
| ***Herpestes javanicus*** | 547.33 ± 41.63 | 85.44 ± 2.45 | 82.67 ± 1.73 | 0.64 ± 0.02 | 487.00 ± 21.70 | 94.55 ± 1.10 | 88.15 ± 1.41 | 0.71 ± 0.02 | 416.33 ± 23.86 | 100.00 ± 0.00 | 99.98 ± 0.01 | 0.45 ± 0.06 | 424.33 ± 30.50 | 93.93 ± 1.02 | 83.60 ± 1.33 | 0.70 ± 0.04 |
| ***Myocastor coypus*** | 442.00 ± 36.50 | 92.49 ± 1.10 | 80.19 ± 1.47 | 0.72 ± 0.01 | 419.67 ± 8.02 | 93.13 ± 0.46 | 81.71 ± 0.27 | 0.73 ± 0.01 | 576.00 ± 17.32 | 99.60 ± 0.10 | 99.84 ± 0.08 | 0.80 ± 0.00 | 474.67 ± 26.69 | 91.95 ± 0.98 | 81.71 ± 1.04 | 0.73 ± 0.00 |
| ***Nasua Nasua*** | 455.67 ± 25.32 | 92.59 ± 1.06 | 75.22 ± 0.58 | 0.68 ± 0.02 | 460.67 ± 99.29 | 95.14 ± 2.34 | 80.85 ± 2.32 | 0.73 ± 0.02 | 422.67 ± 20.50 | 100.00 ± 0.00 | 99.78 ± 0.09 | 0.50 ± 0.07 | 485.67 ± 22.94 | 94.29 ± 0.96 | 80.10 ± 0.93 | 0.72 ± 0.02 |
| ***Nyctereutes procyonides*** | 568.00 ± 33.00 | 92.12 ± 0.75 | 86.03 ± 1.25 | 0.78 ± 0.01 | 478.33 ± 41.19 | 94.89 ± 1.13 | 87.23 ± 0.74 | 0.81 ± 0.01 | 521.67 ± 20.82 | 99.66 ± 0.05 | 99.69 ± 0.01 | 0.84 ± 0.01 | 508.00 ± 75.54 | 95.68 ± 1.11 | 87.93 ± 1.53 | 0.83 ± 0.01 |
| ***Ondatra zibethicus*** | 532.33 ± 41.88 | 82.49 ± 2.19 | 76.73 ± 1.89 | 0.59 ± 0.00 | 427.33 ± 14.36 | 90.18 ± 0.52 | 73.26 ± 0.65 | 0.62 ± 0.01 | 562.67 ± 5.77 | 99.72 ± 0.07 | 99.92 ± 0.02 | 0.70 ± 0.00 | 434.00 ± 9.54 | 89.11 ± 0.62 | 73.24 ± 0.43 | 0.62 ± 0.00 |
| ***Procyon lotor*** | 472.33 ± 11.06 | 88.34 ± 0.52 | 74.91 ± 0.75 | 0.64 ± 0.00 | 542.33 ± 19.55 | 87.43 ± 0.75 | 82.86 ± 1.03 | 0.69 ± 0.01 | 596.00 ± 26.46 | 99.86 ± 0.03 | 99.96 ± 0.03 | 0.84 ± 0.01 | 512.00 ± 25.71 | 87.86 ± 1.28 | 79.60 ± 1.58 | 0.67 ± 0.01 |
| ***Sciurus carolinensis*** | 584.67 ± 5.77 | 90.22 ± 0.46 | 81.89 ± 0.34 | 0.72 ± 0.01 | 551.00 ± 7.00 | 93.59 ± 0.39 | 84.75 ± 0.15 | 0.77 ± 0.01 | 576.00 ± 0.00 | 99.81 ± 0.04 | 99.95 ± 0.01 | 0.89 ± 0.01 | 593.00 ± 20.00 | 90.98 ± 0.86 | 86.28 ± 0.65 | 0.77 ± 0.01 |
| ***Tamias sibiricus*** | 459.00 ± 34.66 | 92.16 ± 1.20 | 72.17 ± 1.43 | 0.62 ± 0.01 | 520.33 ± 55.01 | 91.47 ± 2.27 | 80.31 ± 2.79 | 0.69 ± 0.01 | 441.00 ± 12.12 | 99.98 ± 0.04 | 99.82 ± 0.03 | 0.57 ± 0.03 | 479.00 ± 16.09 | 92.28 ± 0.59 | 79.02 ± 0.93 | 0.71 ± 0.01 |

**Table S6**. Model evaluation metrics for the IAS under study for the different algorithms used (GLM: Generalized linear models, GBM: Boosted regression trees, GAM: Generalized additive models, RF: Random Forest). The metrics displayed correspond to the True Skill Statistic (TSS), including the cutoff values used to transform continuous predictions into binary maps, as well as sensitivity, specificity, and validation results.

**Table S7.** Average importance of explanatory variables in the modeling process for *Herpestes javanicus* (GLM: Generalized linear models, GBM: Boosted regression trees, GAM: Generalized additive models, RF: Random Forest).

|  | **GLM** | **GBM** | **RF** | **GAM** |
| --- | --- | --- | --- | --- |
| **Accessibility** | 0.34 ± 0.01 | 0.34 ± 0.03 | 0.23 ± 0.01 | 0.34 ± 0.04 |
| **bio1** | 1.00 ± 0.00 | 0.44 ± 0.07 | 0.32 ± 0.02 | 0.79 ± 0.01 |
| **bio3** | 0.30 ± 0.04 | 0.16 ± 0.02 | 0.24 ± 0.02 | 0.35 ± 0.03 |
| **bio4** | 0.24 ± 0.01 | 0.03 ± 0.01 | 0.17 ± 0.02 | 0.28 ± 0.01 |
| **bio5** | 0.35 ± 0.02 | 0.02 ± 0.01 | 0.13 ± 0.02 | 0.24 ± 0.02 |
| **bio15** | 0.01 ± 0.01 | 0.06 ± 0.01 | 0.06 ± 0.01 | 0.12 ± 0.00 |
| **bio18** | 0.01 ± 0.00 | 0.01 ± 0.01 | 0.08 ± 0.00 | 0.03 ± 0.01 |
| **bio19** | 0.11 ± 0.01 | 0.07 ± 0.01 | 0.05 ± 0.00 | 0.13 ± 0.01 |
| **Elevation** | 0.14 ± 0.01 | 0.02 ± 0.01 | 0.04 ± 0.00 | 0.15 ± 0.00 |

**Table S8.** Average importance of explanatory variables in the modeling process for *Myocastor coypus* (GLM: Generalized linear models, GBM: Boosted regression trees, GAM: Generalized additive models, RF: Random Forest).

|  | **GLM** | **GBM** | **RF** | **GAM** |
| --- | --- | --- | --- | --- |
| **Accessibility** | 0.18 ± 0.01 | 0.30 ± 0.02 | 0.22 ± 0.01 | 0.15 ± 0.01 |
| **bio1** | 0.14 ± 0.02 | 0.07 ± 0.02 | 0.09 ± 0.01 | 0.19 ± 0.01 |
| **bio3** | 0.03 ± 0.01 | 0.00 ± 0.00 | 0.07 ± 0.00 | 0.05 ± 0.01 |
| **bio4** | 0.29 ± 0.01 | 0.12 ± 0.01 | 0.15 ± 0.00 | 0.37 ± 0.01 |
| **bio5** | 0.01 ± 0.00 | 0.00 ± 0.00 | 0.03 ± 0.00 | 0.02 ± 0.00 |
| **bio15** | 0.08 ± 0.01 | 0.05 ± 0.01 | 0.07 ± 0.01 | 0.04 ± 0.01 |
| **bio18** | 0.00 ± 0.00 | 0.00 ± 0.00 | 0.03 ± 0.00 | 0.01 ± 0.00 |
| **bio19** | 0.00 ± 0.00 | 0.01 ± 0.00 | 0.04 ± 0.00 | 0.01 ± 0.00 |
| **Elevation** | 0.02 ± 0.00 | 0.00 ± 0.00 | 0.02 ± 0.00 | 0.04 ± 0.00 |

**Table S9.** Average importance of explanatory variables in the modeling process for *Nasua nasua* (GLM: Generalized linear models, GBM: Boosted regression trees, GAM: Generalized additive models, RF: Random Forest).

|  | **GLM** | **GBM** | **RF** | **GAM** |
| --- | --- | --- | --- | --- |
| **Accessibility** | 0.03 ± 0.00 | 0.02 ± 0.00 | 0.05 ± 0.00 | 0.05 ± 0.01 |
| **bio1** | 0.24 ± 0.01 | 0.01 ± 0.00 | 0.08 ± 0.01 | 0.51 ± 0.03 |
| **bio3** | 0.27 ± 0.04 | 0.21 ± 0.02 | 0.27 ± 0.01 | 0.35 ± 0.04 |
| **bio4** | 0.17 ± 0.08 | 0.19 ± 0.01 | 0.14 ± 0.01 | 0.24 ± 0.01 |
| **bio5** | 0.05 ± 0.03 | 0.03 ± 0.02 | 0.06 ± 0.00 | 0.17 ± 0.03 |
| **bio15** | 0.05 ± 0.01 | 0.01 ± 0.01 | 0.05 ± 0.01 | 0.05 ± 0.01 |
| **bio18** | 0.02 ± 0.00 | 0.10 ± 0.01 | 0.17 ± 0.01 | 0.08 ± 0.00 |
| **bio19** | 0.00 ± 0.00 | 0.03 ± 0.01 | 0.06 ± 0.00 | 0.04 ± 0.00 |
| **Elevation** | 0.04 ± 0.01 | 0.00 ± 0.00 | 0.04 ± 0.00 | 0.03 ± 0.01 |

**Table S10.** Average importance of explanatory variables in the modeling process for *Nyctereutes procyonoides* (GLM: Generalized linear models, GBM: Boosted regression trees, GAM: Generalized additive models, RF: Random Forest).

|  | **GLM** | **GBM** | **RF** | **GAM** |
| --- | --- | --- | --- | --- |
| **Accessibility** | 0.21 ± 0.01 | 0.32 ± 0.01 | 0.25 ± 0.01 | 0.19 ± 0.01 |
| **bio1** | 0.25 ± 0.01 | 0.08 ± 0.02 | 0.12 ± 0.03 | 0.47 ± 0.02 |
| **bio3** | 0.19 ± 0.02 | 0.18 ± 0.02 | 0.15 ± 0.01 | 0.21 ± 0.01 |
| **bio4** | 0.01 ± 0.01 | 0.04 ± 0.01 | 0.10 ± 0.01 | 0.20 ± 0.01 |
| **bio5** | 0.25 ± 0.03 | 0.02 ± 0.01 | 0.08 ± 0.01 | 0.34 ± 0.04 |
| **bio15** | 0.00 ± 0.00 | 0.00 ± 0.00 | 0.04 ± 0.00 | 0.04 ± 0.01 |
| **bio18** | 0.01 ± 0.00 | 0.06 ± 0.01 | 0.07 ± 0.00 | 0.06 ± 0.01 |
| **bio19** | 0.04 ± 0.00 | 0.01 ± 0.00 | 0.04 ± 0.00 | 0.04 ± 0.01 |
| **Elevation** | 0.02 ± 0.01 | 0.01 ± 0.00 | 0.04 ± 0.00 | 0.02 ± 0.01 |

**Table S11.** Average importance of explanatory variables in the modeling process for *Ondatra zibethicus* (GLM: Generalized linear models, GBM: Boosted regression trees, GAM: Generalized additive models, RF: Random Forest).

|  | **GLM** | **GBM** | **RF** | **GAM** |
| --- | --- | --- | --- | --- |
| **Accessibility** | 0.11 ± 0.01 | 0.23 ± 0.01 | 0.17 ± 0.01 | 0.22 ± 0.02 |
| **bio1** | 0.71 ± 0.05 | 0.18 ± 0.01 | 0.15 ± 0.01 | 0.76 ± 0.02 |
| **bio3** | 0.29 ± 0.03 | 0.04 ± 0.01 | 0.12 ± 0.00 | 0.11 ± 0.01 |
| **bio4** | 0.50 ± 0.04 | 0.02 ± 0.00 | 0.12 ± 0.01 | 0.43 ± 0.04 |
| **bio5** | 0.41 ± 0.03 | 0.00 ± 0.00 | 0.05 ± 0.00 | 0.33 ± 0.02 |
| **bio15** | 0.05 ± 0.00 | 0.05 ± 0.00 | 0.09 ± 0.00 | 0.04 ± 0.01 |
| **bio18** | 0.01 ± 0.00 | 0.01 ± 0.00 | 0.06 ± 0.00 | 0.05 ± 0.00 |
| **bio19** | 0.00 ± 0.00 | 0.02 ± 0.00 | 0.06 ± 0.00 | 0.02 ± 0.01 |
| **Elevation** | 0.04 ± 0.00 | 0.00 ± 0.00 | 0.03 ± 0.00 | 0.03 ± 0.00 |

**Table S12.** Average importance of explanatory variables in the modeling process for *Procyon lotor* (GLM: Generalized linear models, GBM: Boosted regression trees, GAM: Generalized additive models, RF: Random Forest).

|  | **GLM** | **GBM** | **RF** | **GAM** |
| --- | --- | --- | --- | --- |
| **Accessibility** | 0.16 ± 0.01 | 0.34 ± 0.01 | 0.18 ± 0.00 | 0.15 ± 0.00 |
| **bio1** | 0.68 ± 0.02 | 0.03 ± 0.00 | 0.14 ± 0.01 | 0.66 ± 0.03 |
| **bio3** | 0.09 ± 0.01 | 0.06 ± 0.01 | 0.17 ± 0.02 | 0.08 ± 0.02 |
| **bio4** | 0.42 ± 0.01 | 0.02 ± 0.00 | 0.16 ± 0.02 | 0.34 ± 0.01 |
| **bio5** | 0.45 ± 0.01 | 0.00 ± 0.00 | 0.05 ± 0.00 | 0.38 ± 0.01 |
| **bio15** | 0.03 ± 0.00 | 0.05 ± 0.01 | 0.18 ± 0.01 | 0.04 ± 0.00 |
| **bio18** | 0.05 ± 0.01 | 0.01 ± 0.00 | 0.08 ± 0.00 | 0.07 ± 0.01 |
| **bio19** | 0.02 ± 0.00 | 0.07 ± 0.01 | 0.14 ± 0.00 | 0.08 ± 0.01 |
| **Elevation** | 0.00 ± 0.00 | 0.01 ± 0.00 | 0.06 ± 0.00 | 0.03 ± 0.00 |

**Table S13.** Average importance of explanatory variables in the modeling process for *Sciurus carolinensis* (GLM: Generalized linear models, GBM: Boosted regression trees, GAM: Generalized additive models, RF: Random Forest).

|  | **GLM** | **GBM** | **RF** | **GAM** |
| --- | --- | --- | --- | --- |
| **Accessibility** | 0.18 ± 0.01 | 0.31 ± 0.01 | 0.20 ± 0.01 | 0.20 ± 0.01 |
| **bio1** | 0.35 ± 0.02 | 0.02 ± 0.00 | 0.10 ± 0.01 | 0.39 ± 0.01 |
| **bio3** | 0.22 ± 0.00 | 0.09 ± 0.01 | 0.11 ± 0.00 | 0.20 ± 0.01 |
| **bio4** | 0.10 ± 0.01 | 0.01 ± 0.00 | 0.09 ± 0.01 | 0.21 ± 0.02 |
| **bio5** | 0.22 ± 0.01 | 0.00 ± 0.00 | 0.03 ± 0.00 | 0.35 ± 0.01 |
| **bio15** | 0.06 ± 0.01 | 0.11 ± 0.00 | 0.20 ± 0.01 | 0.12 ± 0.01 |
| **bio18** | 0.07 ± 0.01 | 0.00 ± 0.00 | 0.07 ± 0.00 | 0.09 ± 0.01 |
| **bio19** | 0.03 ± 0.00 | 0.07 ± 0.00 | 0.13 ± 0.01 | 0.05 ± 0.00 |
| **Elevation** | 0.01 ± 0.00 | 0.03 ± 0.00 | 0.07 ± 0.00 | 0.02 ± 0.00 |

**Table S14.** Average importance of explanatory variables in the modeling process for *Tamias sibiricus* (GLM: Generalized linear models, GBM: Boosted regression trees, GAM: Generalized additive models, RF: Random Forest).

|  | **GLM** | **GBM** | **RF** | **GAM** |
| --- | --- | --- | --- | --- |
| **Accessibility** | 0.27 ± 0.01 | 0.25 ± 0.01 | 0.27 ± 0.02 | 0.26 ± 0.01 |
| **bio1** | 0.47 ± 0.01 | 0.01 ± 0.00 | 0.08 ± 0.00 | 0.42 ± 0.01 |
| **bio3** | 0.07 ± 0.01 | 0.37 ± 0.02 | 0.13 ± 0.01 | 0.21 ± 0.04 |
| **bio4** | 0.82 ± 0.01 | 0.08 ± 0.01 | 0.12 ± 0.01 | 0.75 ± 0.07 |
| **bio5** | 0.92 ± 0.02 | 0.11 ± 0.02 | 0.10 ± 0.01 | 0.71 ± 0.10 |
| **bio15** | 0.02 ± 0.01 | 0.02 ± 0.01 | 0.07 ± 0.01 | 0.08 ± 0.02 |
| **bio18** | 0.04 ± 0.00 | 0.09 ± 0.01 | 0.17 ± 0.01 | 0.09 ± 0.01 |
| **bio19** | 0.03 ± 0.00 | 0.00 ± 0.01 | 0.03 ± 0.00 | 0.05 ± 0.01 |
| **Elevation** | 0.06 ± 0.01 | 0.00 ± 0.01 | 0.02 ± 0.00 | 0.02 ± 0.01 |

**Table S15**. Average Importance of Explanatory Variables for All Species. (GLM: Generalized Linear Models, GBM: Boosted Regression Trees, GAM: Generalized Additive Models, RF: Random Forest).

|  | **GLM** | **GBM** | **RF** | **GAM** |
| --- | --- | --- | --- | --- |
| **Accessibility** | 0.18 ± 0.09 | 0.26 ± 0.11 | 0.20 ± 0.07 | 0.19 ± 0.08 |
| **bio1** | 0.48 ± 0.29 | 0.11 ± 0.15 | 0.13 ± 0.08 | 0.52 ± 0.20 |
| **bio3** | 0.18 ± 0.11 | 0.14 ± 0.12 | 0.16 ± 0.07 | 0.20 ± 0.11 |
| **bio4** | 0.32 ± 0.26 | 0.06 ± 0.06 | 0.13 ± 0.03 | 0.35 ± 0.18 |
| **bio5** | 0.33 ± 0.28 | 0.02 ± 0.04 | 0.07 ± 0.03 | 0.32 ± 0.20 |
| **bio15** | 0.04 ± 0.03 | 0.04 ± 0.04 | 0.10 ± 0.06 | 0.07 ± 0.04 |
| **bio18** | 0.03 ± 0.02 | 0.04 ± 0.04 | 0.09 ± 0.05 | 0.06 ± 0.03 |
| **bio19** | 0.03 ± 0.04 | 0.04 ± 0.03 | 0.07 ± 0.04 | 0.05 ± 0.04 |
| **Elevation** | 0.04 ± 0.04 | 0.01 ± 0.01 | 0.04 ± 0.02 | 0.04 ± 0.04 |

**
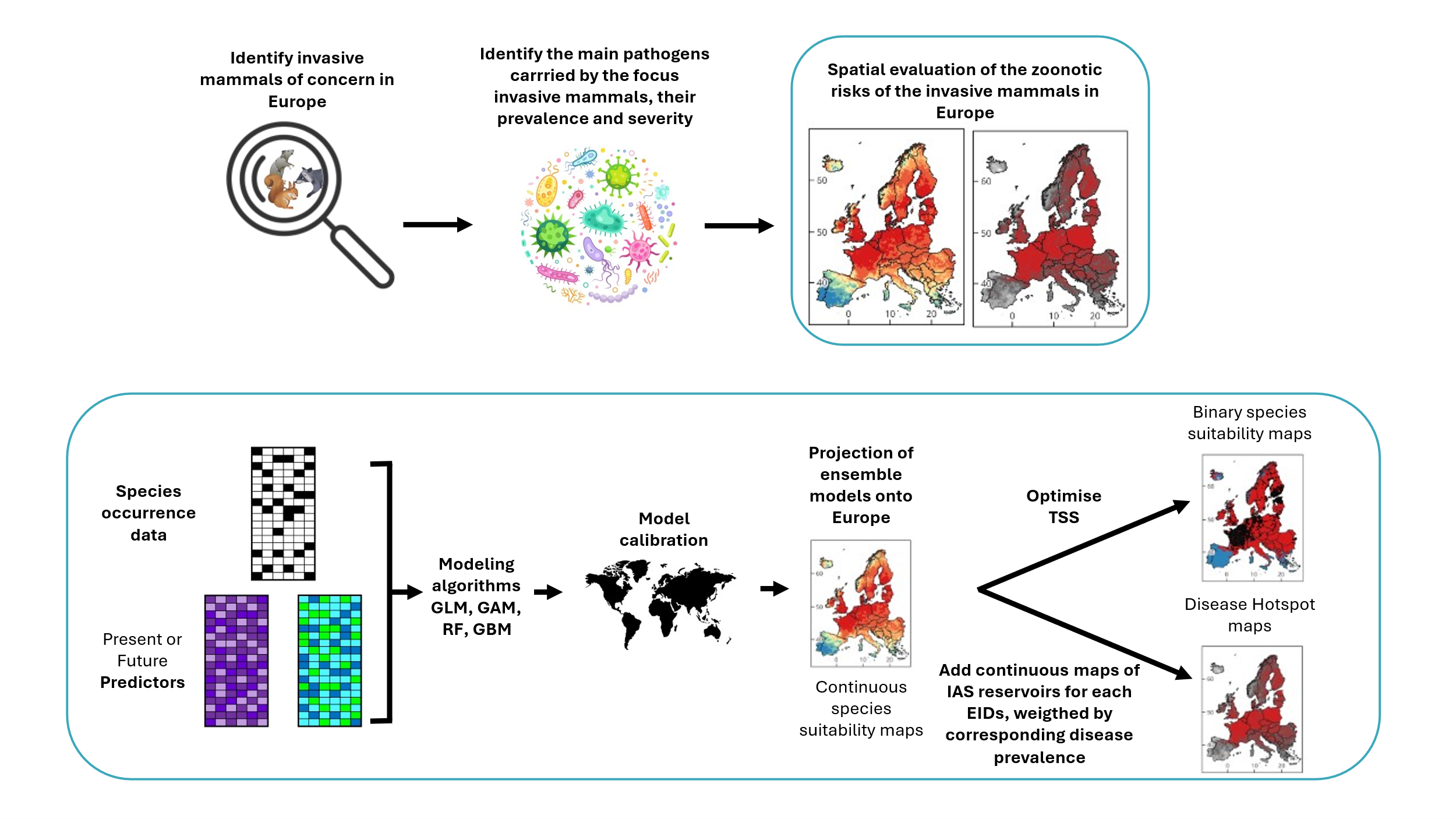
Figure S1.** Graphical abstract.


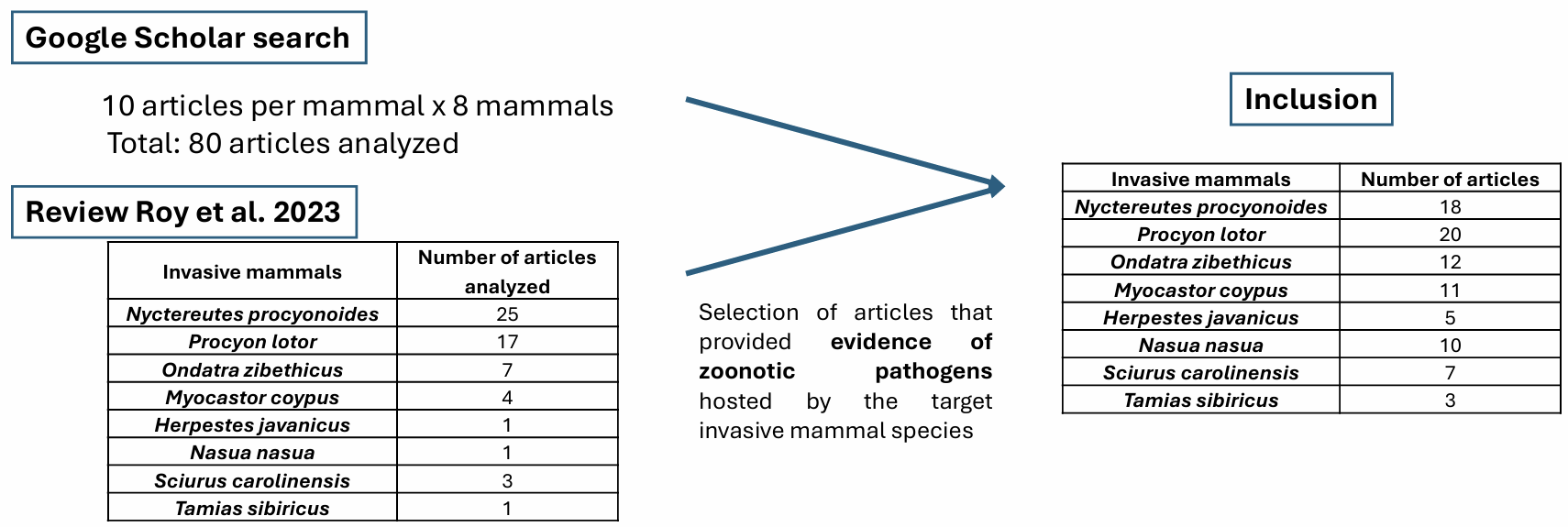


**Figure S2.** Overview of the article selection process identifying studies with evidence of zoonotic pathogens for the 8 selected invasive mammals.

**Figure S3.** Importance of variables in distribution models for eight invasive mammals. Data corresponds to the average of the eight species investigated. Variable acronyms can be consulted in Table S2.

**Figure S4.** Importance of variables in species distribution models. Data corresponds to the average of the eight species investigated. Variable acronyms can be consulted in Table S2.


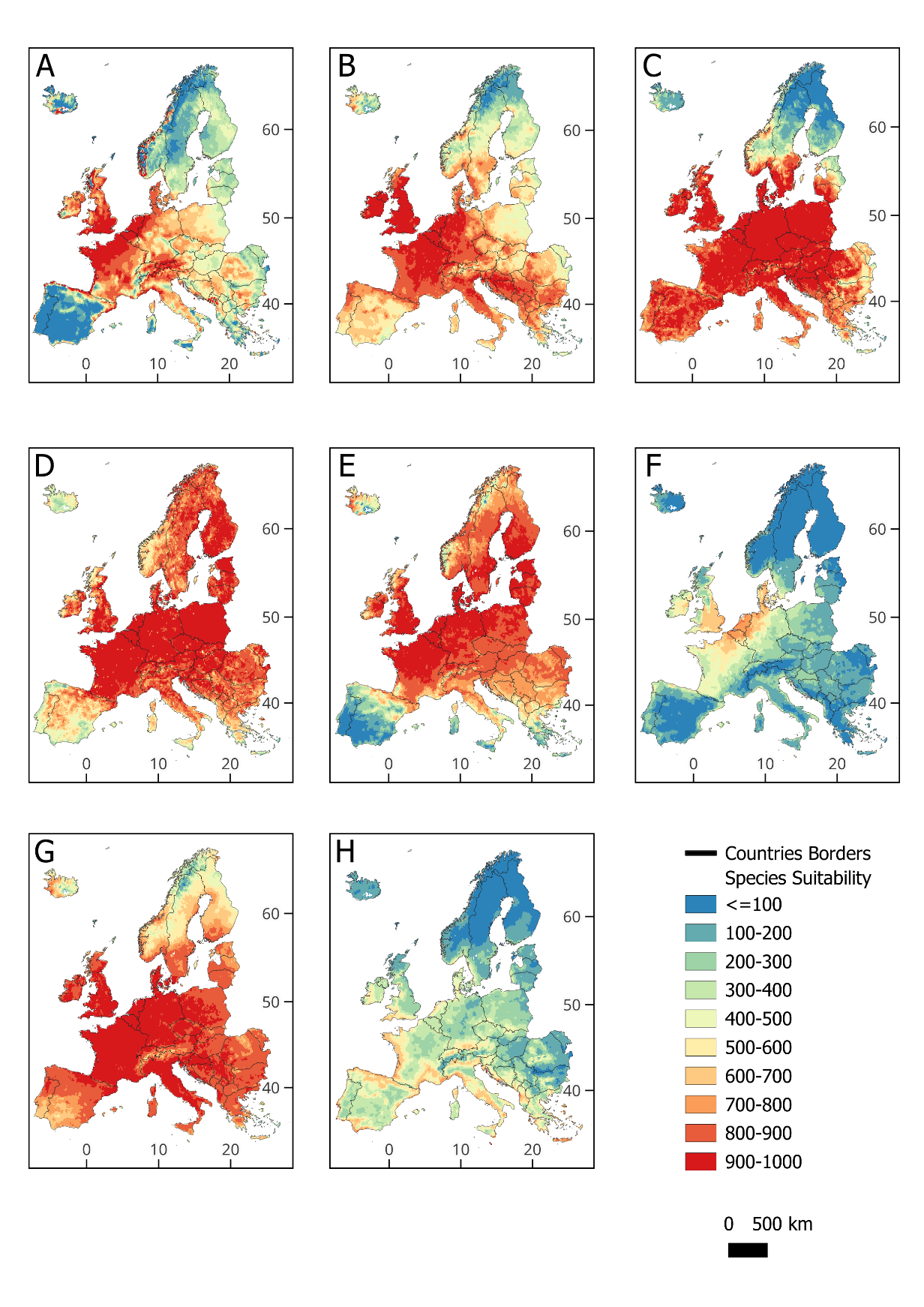


**Figure S5.** Continuous maps displaying the current predicted suitability for eight invasive mammals regulated in Europe. Species depicted: A. *Tamias sibiricus*, B. *Sciurus carolinensis*, C. *Procyon lotor*, D. *Ondatra zibethicus*, E. *Nyctereutes procyonoides*, F. *Nasua nasua*, G. *Myocastor coypus*, H. *Herpestes javanicus*. Warmer colors (e.g., red and orange) indicate areas of higher habitat suitability, while cooler colors (e.g., blue and green) represent lower suitability levels


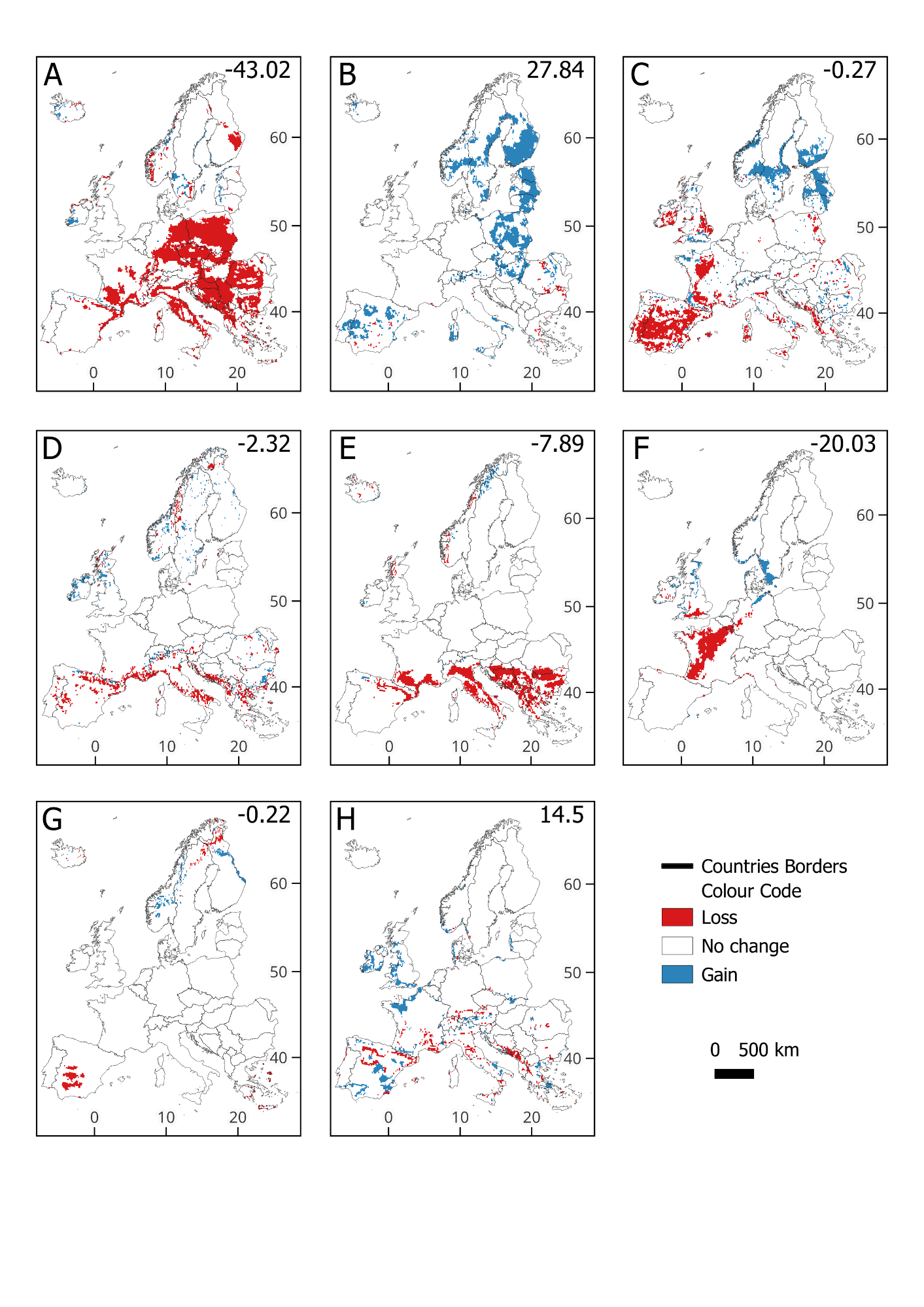


**Figure S6.** Maps displaying changes in the future suitability for IAS in Europe under the Business As Usual (BAU) scenario compared to the current scenario. Species depicted: A. *Tamias sibiricus*, B. *Sciurus carolinensis*, C. *Procyon lotor*, D. *Ondatra zibethicus*, E. *Nyctereutes procyonoides*, F. *Nasua nasua*, G. *Myocastor coypus*, H. *Herpestes javanicus*. In the top right corner, we display the species range change (SRC) as the percentage area gained or lost by 2050 relative to the current scenario.


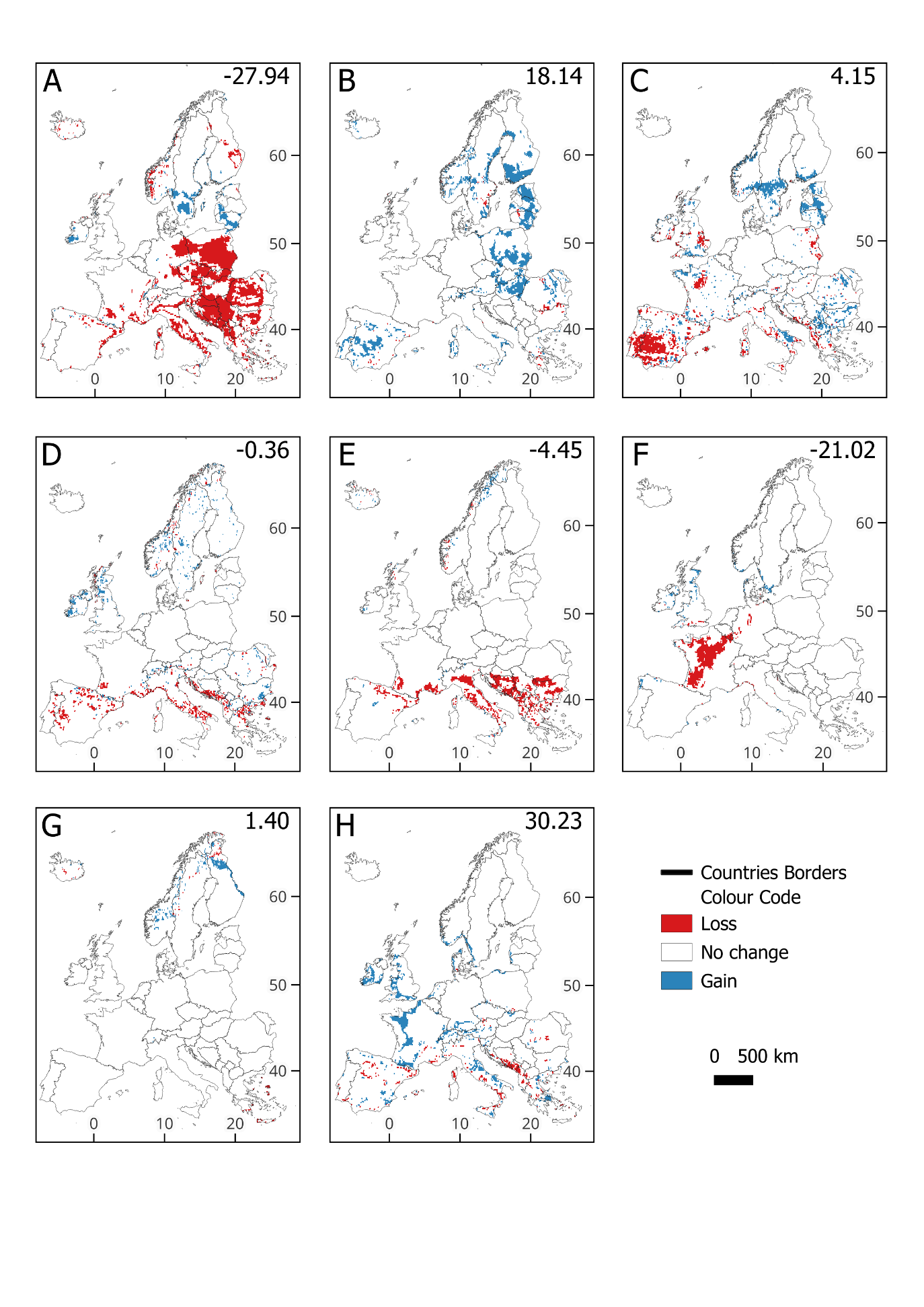


**Figure S7.** Maps displaying changes in the future suitability for IAS in Europe under the Low Emissions scenario compared to the current scenario. Species depicted: A. *Tamias sibiricus*, B. *Sciurus carolinensis*, C. *Procyon lotor*, D. *Ondatra zibethicus*, E. *Nyctereutes procyonoides*, F. *Nasua nasua*, G. *Myocastor coypus*, H. *Herpestes javanicus*. In the top right corner, we display the species range change (SRC) as the percentage area gained or lost by 2050 relative to the current scenario.

## REFERENCES LITERATURE REVIEW

1. Rhynd, K. J. R. et al. Prevalence of *Salmonella* spp. and thermophilic *Campylobacter* spp. in the small asian mongoose (*Herpestes Javanicus*) in Barbados, west indies. Journal of Zoo and Wildlife Medicine 45, 911–914 (2014).

2. Li, T. C. et al. Serologic evidence for *Hepatitis E virus* infection in mongoose. Am J Trop Med Hyg 74, 932–936 (2006).

3. Saito, M. et al. Proposal for Japanese Encephalitis Surveillance Using Captured Invasive Mongooses Under an Eradication Project on Okinawa Island, Japan. Vector-Borne and Zoonotic Diseases, 9, 259–266 (2009).

4. Shiokawa, K. et al. Peridomestic small Indian mongoose: An invasive species posing as potential zoonotic risk for leptospirosis in the Caribbean. Acta Trop 190, 166–170 (2019).

5. Nakamura, M. et al. *Hepatitis E virus* infection in wild mongooses of Okinawa, Japan: Demonstration of anti-HEV antibodies and a full-genome nucleotide sequence. Hepatology Research 34, 137–140 (2006).

6. Liu, X. et al. Occurrence and subtyping of *Blastocystis* in coypus (*Myocastor coypus*) in China. Parasit Vectors 15, 14 (2022).

7. Lim, S. R. et al. Wild Nutria (*Myocastor coypus*) Is a Potential Reservoir of Carbapenem-Resistant and Zoonotic *Aeromonas* spp. in Korea. Microorganisms 7, 224 (2019).

8. Martino, P. E. et al. Seroprevalence for selected pathogens of zoonotic importance in wild nutria (*Myocastor coypus*). Eur J Wildl Res 60, 551–554 (2014).

9. Cui, Z. et al. Occurrence and Multi-Locus Analysis of *Giardia duodenalis* in Coypus (*Myocastor coypus*) in China. Pathogens 10, 179 (2021).

10. Gayo, V. et al. Natural *Fasciola hepatica* Infection in Nutria (*Myocastor coypus*) in Uruguay. Journal of Zoo and Wildlife Medicine 42, 354–356 (2011).

11. Yu, F. et al. Host-adaptation of the rare *Enterocytozoon bieneusi* genotype CHN4 in *Myocastor coypus* (Rodentia: Echimyidae) in China. Parasit Vectors 13, 1–8 (2020).

12. Zanzani, S. A. et al. Parasitic and Bacterial Infections of *Myocastor coypus* in a Metropolitan Area of Northwestern Italy. J Wildl Dis 52, 126–130 (2016).

13. Park, S. Y. et al. Complete Genome Sequence of *Aeromonas rivipollensis* KN-Mc-11N1, Isolated from a Wild Nutria (*Myocastor coypus*) in South Korea. Microbiol Resour Announc 7, (2018).

14. Nardoni, S., Angelici, M. C., Mugnaini, L. & Mancianti, F. Prevalence of *Toxoplasma gondii* infection in *Myocastor coypus* in a protected Italian wetland. Parasit Vectors 4, 1–4 (2011).

15. Umhang, G., Richomme, C., Boucher, J. M., Guedon, G. & Boué, F. Nutrias and muskrats as bioindicators for the presence of *Echinococcus multilocularis* in new endemic areas. Vet Parasitol 197, 283–287 (2013).

16. Asakawa, M., Sato, M., Sone, K., Tatsuzawa, S. & Oda, S. Further helminthological survey on alien rodents, copyu (*Myocastor coypus*: Myocastoridae) in Aichi and Hyogo Prefecture, Japan. Journal of Rakuno Gakuen University, Natural Science 33, 291–292 (2009).

17. Moraes, M. F. D. et al. Filarial nematodes with zoonotic potential in ring-tailed coatis (*Nasua nasua* Linnaeus, 1766, Carnivora: Procyonidae) and domestic dogs from Iguaçu National Park, Brazil. Vet Parasitol Reg Stud Reports 8, 1–9 (2017).

18. Silva, R. O. S. et al. Carriage of *Clostridium difficile* in free-living South American coati (*Nasua nasua*) in Brazil. Anaerobe 30, 99–101 (2014).

19. Guimarães, F. de R. et al. Parasitism of *Dirofilaria incrassata* Molin, 1858 in coatis (*Nasua nasua*). Vet Parasitol Reg Stud Reports 39, 100842 (2023).

20. Alves, F. M. et al. Modulating Variables of *Trypanosoma cruzi* and *Trypanosoma evansi* Transmission in Free-Ranging Coati (*Nasua nasua*) from the Brazilian Pantanal Region. Vector-Borne and Zoonotic Diseases 11, 835–841 (2011).

21. Perles, L. et al. Co-infection by multiple vector-borne agents in wild ring-tailed coatis (*Nasua nasua*) from Iguaçu National Park, southern Brazil. Scientific Reports 13, 1828 (2023).

22. Cubilla, M. P. et al. Microscopic and molecular identification of hemotropic mycoplasmas in South American coatis (*Nasua nasua*). Comp Immunol Microbiol Infect Dis 53, 19–25 (2017).

23. Almeida, L. R. et al. *Angiostrongylus minasensis* n. sp.: new species found parasitizing coatis (*Nasua nasua*) in an urban protected area in Brazil. Revista Brasileira de Parasitologia Veterinária 29, e018119 (2020).

24. Perles, L. et al. Molecular detection of *Babesia* spp. and *Rickettsia* spp. in coatis (*Nasua nasua*) and associated ticks from midwestern Brazil. Parasitol Res 122, 1151–1158 (2023).

25. Mehrkens, L. R. et al. White-nosed coatis (*Nasua narica*) are a potential reservoir of T*rypanosoma cruzi* and other potentially zoonotic pathogens in Monteverde, Costa Rica. J Wildl Dis 49, 1014–1018 (2013).

26. Davidson, R. K., Øines, Ø., Hamnes, I. S. & Schulze, J. E. Illegal Wildlife Imports More than Just Animals—*Baylisascaris procyonis* in Raccoons (*Procyon lotor*) in Norway. J Wildl Dis 49, 986–990 (2013).

27. Kjær, L. J., Jensen, L. M., Chriél, M., Bødker, R. & Petersen, H. H. The raccoon dog (*Nyctereutes procyonoides*) as a reservoir of zoonotic diseases in Denmark. Int J Parasitol Parasites Wildl 16, 175–182 (2021).

28. Duscher, T., Hodžić, A., Glawischnig, W. & Duscher, G. G. The raccoon dog (*Nyctereutes procyonoides*) and the raccoon (*Procyon lotor*)—their role and impact of maintaining and transmitting zoonotic diseases in Austria, Central Europe. Parasitol Res 116, 1411–1416 (2017).

29. Lempp, C. et al. Pathological findings in the red fox (*Vulpes vulpes*), stone marten (*Martes foina*) and raccoon dog (*Nyctereutes procyonoides*), with special emphasis on infectious and zoonotic agents in Northern Germany. PLoS One 12, e0175469 (2017).

30. Yang, Y. et al. Widespread presence of human-pathogenic *Enterocytozoon bieneusi* genotype D in farmed foxes (*Vulpes vulpes*) and raccoon dogs (*Nyctereutes procyonoides*) in China: first identification and zoonotic concern. Parasitol Res 114, 4341–4348 (2015).

31. Zhao, W. et al. Genotyping of *Enterocytozoon bieneusi* in Farmed Blue Foxes (*Alopex lagopus*) and Raccoon Dogs (*Nyctereutes procyonoides*) in China. PLoS One 10, e0142611 (2015).

32. Karamon, J. et al. G Intestinal helminths of raccoon dogs (*Nyctereutes procyonoides*) and red foxes (*Vulpes vulpes*) from the Augustów Primeval Forest (north-eastern Poland). J Vet Res 60, 273–277 (2016).

33. Zhang, S. et al. First report of *Cryptosporidium canis* in foxes (*Vulpes vulpes*) and raccoon dogs (*Nyctereutes procyonoides*) and identification of several novel subtype families for *Cryptosporidium mink* genotype in minks (Mustela vison) in China. Infection, Genetics and Evolution 41, 21–25 (2016).

34. Bružinskaite-Schmidhalter, R. et al. Helminths of red foxes (*Vulpes vulpes*) and raccoon dogs (*Nyctereutes procyonoides*) in Lithuania. Parasitology 139, 120–127 (2012).

35. Al-Sabi, M. N. S., Chriél, M., Jensen, T. H. & Enemark, H. L. Endoparasites of the raccoon dog (*Nyctereutes procyonoides*) and the red fox (*Vulpes vulpes*) in Denmark 2009–2012 – A comparative study. Int J Parasitol Parasites Wildl 2, 144–151 (2013).

36. Laurimaa, L. et al. Alien species and their zoonotic parasites in native and introduced ranges: The raccoon dog example. Vet Parasitol 219, 24–33 (2016).

37. Härtwig, V. et al. Detection of *Anaplasma phagocytophilum* in red foxes (*Vulpes vulpes*) and raccoon dogs (*Nyctereutes procyonoides*) from Brandenburg, Germany. Ticks Tick Borne Dis 5, 277–280 (2014).

38. Schwarz, S. et al. Estimated prevalence of *Echinococcus multilocularis* in raccoon dogs *Nyctereutes procyonoides* in northern Brandenburg, Germany. Curr Zool 57, 655–661 (2011).

39. Laurimaa, L. et al. First report of the zoonotic tapeworm *Echinococcus multilocularis* in raccoon dogs in Estonia, and comparisons with other countries in Europe. Vet Parasitol 212, 200–205 (2015).

40. Kuehn, A. et al. Tularaemia seroprevalence of captured and wild animals in Germany: the fox (*Vulpes vulpes*) as a biological indicator. Epidemiol Infect 141, 833–840 (2013).

41. Zienius, D., Bagdonas, J. & Dranseika, A. Epidemiological situation of rabies in Lithuania from 1990 to 2000. Vet Microbiol 93, 91–100 (2003).

42. Cybulska, A., Kornacka, A. & Moskwa, B. The occurrence and muscle distribution of *Trichinella britovi* in raccoon dogs (*Nyctereutes procyonoides*) in wildlife in the Głęboki Bród Forest District, Poland. Int J Parasitol Parasites Wildl 9, 149–153 (2019).

43. Mayer-Scholl, A., Reckinger, S., Schulze, C. & Nöckler, K. Study on the occurrence of *Trichinella* spp. in raccoon dogs in Brandenburg, Germany. Vet Parasitol 231, 102–105 (2016).

44. Maèiulskis, P., Lukauskas, K., Sederevièius, A., Kiudulas, V. & Pockevièius, A. Epidemiology of enzootic rabies in Lithuania. Med Weter 62, 769–772 (2006).

45. Lombardo, A. et al. First report of the zoonotic nematode *Baylisascaris procyonis* in non-native raccoons (*Procyon lotor*) from Italy. Parasit Vectors 15, 1–5 (2022).

46. Mohammad Rahimi, H. et al. Molecular characterization of *Cryptosporidium skunk* genotype in raccoons (*Procyon lotor*) in Iran: concern for zoonotic transmission. Parasitol Res 121, 483–489 (2022).

47. Solarczyk, P. et al. Zoonotic *Giardia duodenalis* sub-assemblage BIV in wild raccoons (*Procyon loto*r) from Germany and Luxembourg. Zoonoses Public Health 68, 538–543 (2021).

48. Sandfort, R. F., Murray, W. & Janda, J. M. *Moellerella wisconsensis* Isolated from the Oral Cavity of a Wild Raccoon (*Procyon lotor*). Vector Borne and Zoonotic Diseases 2, 197–199 (2002).

49. Mohammad Rahimi, H., Nemati, S., Mirjalali, H., Sharifdini, M. & Zali, M. R. Molecular characterization and identification of *Blastocystis* and its subtypes from raccoon (*Procyon lotor*) in north of Iran. Parasitol Res 119, 2741–2745 (2020).

50. Rentería-Solís, Z., Birka, S., Schmäschke, R., Król, N. & Obiegala, A. First detection of *Baylisascaris procyonis* in wild raccoons (*Procyon lotor*) from Leipzig, Saxony, Eastern Germany. Parasitol Res 117, 3289–3292 (2018).

51. Duncan, C. et al. Leptospirosis and Tularaemia in Raccoons (*Procyon lotor*) of Larimer Country, Colorado. Zoonoses Public Health 59, 29–34 (2012).

52. Stope, M. Wild raccoons in Germany as a reservoir for zoonotic agents. Eur J Wildl Res 65, 1–7 (2019).

53. Al-Sabi, M. N. S., Chriél, M., Hansen, M. S. & Enemark, H. L. *Baylisascaris procyonis* in wild raccoons (*Procyon lotor*) in Denmark. Vet Parasitol Reg Stud Reports 1–2, 55–58 (2015).

54. Bauer, C. Baylisascariosis (*Baylisascaris procyonis*)--a rare parasitic zoonosis in Europe. Berl Munch Tierarztl Wochenschr 124, 465–472 (2011).

55. Sato, H., Kamiya, H. & Furuoka, H. Epidemiological Aspects of the First Outbreak of *Baylisascaris procyonis* Larva Migrans in Rabbits in Japan. Journal of Veterinary Medical Science 65, 453–457 (2003).

56. Inoue, K. et al. Serological Survey of Five Zoonoses, Scrub Typhus, Japanese Spotted Fever, Tularemia, Lyme Disease, and Q Fever, in Feral Raccoons (*Procyon lotor*) in Japan. Vector-Borne and Zoonotic Diseases 11, 15–19 (2011).

57. Karamon, J., Kochanowski, M., Cencek, T., Bartoszewicz, M. & Kusyk, P. Gastrointestinal helminths of raccoons (*Procyon lotor*) in western Poland (lubuskie province)-with particular regard to *Baylisascaris procyonis*. J Vet Res 58, 547–552 (2014).

58. Leśniańska, K. et al. *Cryptosporidium* spp. and *Enterocytozoon bieneusi* in introduced raccoons (*Procyon lotor*)—first evidence from Poland and Germany. Parasitol Res 115, 4535–4541 (2016).

59. Stolte, M., Odentng, K., Walter, G. & Bockhardt, I. The Raccoon as Intermediate Host of Three Sarcocystis Species in Europe. J. Helminthol. Soc. Wash. 63, 145–149 (1996).

60. Sato, H., Suzoki, K., Osanai, A., Kamiya, H. & Furuoka, H. Identification and characterization of the threadworm, *Strongyloides procyonis*, from feral raccoons (*Procyon lotor*) in Japan. Journal of Parasitology 92, 63–68 (2006).

61. Davidson, R. K., Øines, Ø., Hamnes, I. S. & Schulze, J. E. Illegal Wildlife Imports More than Just Animals—*Baylisascaris procyonis* in Raccoons (*Procyon lotor*) in Norway. J Wildl Dis 49, 986–990 (2013).

62. Lee, K. et al. Prevalence of *Salmonella*, *Yersinia* and *Campylobacter* spp. in Feral Raccoons (*Procyon lotor*) and Masked Palm Civets (*Paguma larvata*) in Japan. Zoonoses Public Health 58, 424–431 (2011).

63. Nowakiewicz, A. et al. Free-Living Species of Carnivorous Mammals in Poland: Red Fox, Beech Marten, and Raccoon as a Potential Reservoir of *Salmonella*, *Yersinia*, *Listeria* spp. and Coagulase-Positive *Staphylococcus*. PLoS One 11, e0155533 (2016).

64. Greenwood, A. G. & Sanchez, S. Serological evidence of murine pathogens in wild grey squirrels (*Sciurus carolinensis*) in North Wales. Veterinary Record 150, 543–546 (2002).

65. Bown, K. J. et al. New World origins for haemoparasites infecting United Kingdom grey squirrels (*Sciurus carolinensis*), as revealed by phylogenetic analysis of *Bartonella* infecting squirrel populations in England and the United States. Epidemiol Infect 129, 647–653 (2002).

66. Gurnell, J., Lurz, P. W. & Shuttleworth, C. M. Ecosystem impacts of an alien invader in Europe, the grey squirrel *Sciurus carolinensis*. (2016).

67. Cruciani, D. et al. Health Status of the Eastern Grey Squirrel (*Sciurus carolinensis*) Population in Umbria: Results of the LIFE Project ‘U-SAVEREDS’. Animals 12, 2741 (2022).

68. Millins, C. et al. An invasive mammal (the gray squirrel, *Sciurus carolinensis*) commonly hosts diverse and atypical genotypes of the zoonotic pathogen *Borrelia burgdorferi* Sensu lato. Appl Environ Microbiol 81, 4236–4245 (2015).

69. Romeo, C. et al. Are tree squirrels involved in the circulation of flaviviruses in Italy? Transbound Emerg Dis 65, 1372–1376 (2018).

70. Schlottau, K. et al. Multiple detection of zoonotic *variegated squirrel bornavirus 1* RNA in different squirrel species suggests a possible unknown origin for the virus. Arch Virol 162, 2747–2754 (2017).

71. Rar, V. A. et al. Genetic Variability of *Anaplasma phagocytophilum* in *Ixodes persulcatus* Ticks and Small Mammals in the Asian Part of Russia. Vector-Borne and Zoonotic Diseases 11, 1013–1021 (2011).

72. Marsot, M. et al. Introduced Siberian chipmunks (*Tamias sibiricus* barberi) harbor more-diverse *Borrelia burgdorferi* sensu lato genospecies than native bank voles (*Myodes glareolus*). Appl Environ Microbiol 77, 5716–5721 (2011).

73. Vourc’h, G., Marmet, J., Chassagne, M., Bord, S. & Chapuis, J. L. *Borrelia burgdorferi* Sensu Lato in Siberian Chipmunks (*Tamias sibiricus*) Introduced in Suburban Forests in France. Vector-Borne and Zoonotic Diseases 7, 637–641 (2007).

74. Schuster, R. K., Specht, P. & Rieger, S. On the Helminth Fauna of the Muskrat (*Ondatra zibethicus* (Linnaeus, 1766)) in the Barnim District of Brandenburg State/Germany. Animals 11, 2444 (2021).

75. Krügel, M. et al. Rats as potential reservoirs for neglected zoonotic *Bartonella* species in Flanders, Belgium. Parasit Vectors 13, 235 (2020).

76. Ganoe, L. S. et al. Surveillance for diseases, pathogens, and toxicants of muskrat (*Ondatra zibethicus*) in Pennsylvania and surrounding regions. PLoS One 16, e0260987 (2021).

77. Niedringhaus, K. D. et al. Fatal infection with *Versteria* sp. in a muskrat, with implications for human health. Journal of Veterinary Diagnostic Investigation 34, 314–318 (2022).

78. Ganoe, L. S., Brown, J. D., Yabsley, M. J., Lovallo, M. J. & Walter, W. D. A Review of Pathogens, Diseases, and Contaminants of Muskrats (*Ondatra zibethicus*) in North America. Front Vet Sci 7, 233 (2020).

79. Li, J., Qin, H., Li, X. & Zhang, L. Role of rodents in the zoonotic transmission of giardiasis. One Health 100500 (2023) doi:10.1016/J.ONEHLT.2023.100500.

80. Hanosset, R., Saegerman, C., Adant, S., Massart, L. & Losson, B. *Echinococcus multilocularis* in Belgium: Prevalence in red foxes (*Vulpes vulpes*) and in different species of potential intermediate hosts. Vet Parasitol 151, 212–217 (2008).

81. Baumeister, S., Pohlmeyer, K., Kuschfeldt, S. & Stoye, M. Prevalence of *Echinococcus multilocularis* and other metacestodes and cestodes in the muskrat (*Ondatra zibethicus* LINK 1795) in Lower Saxony. Dtsch Tierarztl Wochenschr 104, 448–452 (1997).

82. Karanis, P., Opiela, K., Renoth, S. & Seitz, H. M. Possible Contamination of Surface Waters with *Giardia* spp. through Muskrats. Zentralblatt für Bakteriologie 284, 302–306 (1996).

83. Vahlenkamp, M. et al. The muskrat (*Ondatra zibethicus*) as a new reservoir for puumala-like *hantavirus* strains in Europe. Virus Res 57, 139–150 (1998).

84. Lataste-Dorolle, C. & Fiocre, B. Muskrats, *Ondatra* (*Fiber zibethicus*, Linnaeus), carriers in France of various leptospirian serotypes: isolation of the 1st French strain related to the Hebdomadis serogroup. Bull Soc Pathol Exot Filiales 62, 312–320 (1969).
